# CGR11 promotes hepatocellular carcinoma progression by regulating autophagy through the PI3K/AKT pathway

**DOI:** 10.3389/fcell.2025.1692480

**Published:** 2026-01-07

**Authors:** Jia Zhou, Sulai Liu, Yinghui Song, Junjie Liu, Zhiguo Tan, Jie Liu, Xiaoxia Han, Yang Xing, Xinrun Wang, Chuang Peng, Bo Sun, Yufang Leng

**Affiliations:** 1 The First School of Clinical Medicine, Lanzhou University, Lanzhou, China; 2 Department of Hepatobiliary Surgery, Hunan Provincial People’s Hospital (The First Affiliated Hospital of Hunan Normal University), Changsha, China; 3 Department of General Surgery, Hunan Aerospace Hospital, Changsha, China; 4 Department of Anesthesiology, The First Hospital of Lanzhou University, Lanzhou, China

**Keywords:** autophagy, CGR11, hepatocellular carcinoma, molecular mechanism, PI3K/AKT

## Abstract

**Background:**

Hepatocellular carcinoma (HCC), the predominant pathological subtype of primary liver cancer, remains a major global health burden with poorly defined molecular mechanisms. Cell growth regulator 11 (CGR11), a novel secreted protein characterized by EF-hand motifs, has recently emerged as a potential extracellular signaling modulator in tumor biology. Although implicated in cancer cell proliferation and metastasis, its precise role and regulatory mechanisms in HCC progression have not been elucidated.

**Methods:**

We integrated bioinformatics analysis with single-cell transcriptomic profiling and CellChat-based intercellular communication mapping. CGR11 expression and localization were validated in tissue microarrays, HCC cell lines, and tumor specimens using immunohistochemical staining, qRT-PCR, and Western blotting. *In vitro* experiments and both subcutaneous and orthotopic xenograft models were established to evaluate the biological effects of CGR11 overexpression and knockdown. RNA sequencing, LC3 fluorescence assay, and transmission electron microscopy were conducted to elucidate the underlying molecular mechanism.

**Results:**

CGR11 expression was markedly increased in HCC tissues relative to adjacent non-tumorous liver tissues and correlated with poor patient prognosis. Functional and mechanistic analyses demonstrated that CGR11 promotes HCC cell proliferation, invasion and tumor growth by inhibiting autophagy levels through activation of the PI3K/AKT signaling. Conversely, CGR11 knockdown restored autophagy and significantly suppressed tumor progression in both cellular and animal models.

**Conclusion:**

Our findings establish CGR11 as a novel oncogenic regulator that contributes to HCC progression by suppressing autophagy via PI3K/AKT activation. Targeting the CGR11-PI3K/AKT axis may therefore provide a promising avenue for precision therapeutic intervention in HCC.

## 1 Introduction

Primary liver cancer remains a major global health burden, ranking as the sixth most commonly diagnosed malignancy and the fourth leading cause of cancer-related mortality worldwide (Sung et al., 2021; Xia et al., 2022). Hepatocellular carcinoma (HCC), which accounts for 75%–85% of primary liver cancers, represents a highly aggressive malignancy that contributes disproportionately to cancer-related deaths, particularly in China (Sung et al., 2021; Wang et al., 2024). Despite significant advances in therapeutic modalities including surgical resection, liver transplantation, molecular targeted therapies, and immune checkpoint inhibitors, the prognosis for advanced HCC remains poor, with 5-year survival rates persistently below 20% (Villanueva, 2019; Llovet et al., 2021). The limited efficacy of current treatments is largely attributed to profound molecular heterogeneity, adaptive drug resistance, and the complex tumor microenvironment, highlighting an urgent need to identify novel molecular targets and clarify the signaling mechanisms that drive HCC progression and therapy resistance (Sonkin et al., 2024). HCC pathogenesis is governed by intricate molecular networks involving oncogenic signaling, metabolic reprogramming, and dysregulated autophagy (Xu et al., 2025). Among these, the phosphatidylinositol 3-kinase (PI3K)/AKT/mTOR pathway plays a central role in regulating cell proliferation, survival, and metabolism (Tian et al., 2023). Hyperactivation of this pathway suppresses autophagy initiation through inhibition of the ULK1/2 complex and PTEN inactivation, leading to enhanced tumor growth and resistance to apoptosis (Wang et al., 2018; Quan et al., 2025). Moreover, crosstalk between autophagy and the PI3K/AKT axis contributes to malignant phenotypes such as epithelial–mesenchymal transition (EMT), immune evasion, and remodeling of the tumor microenvironment (Zhang et al., 2019; Peng et al., 2020; Huang et al., 2022). Despite these insights, the upstream regulatory factors that modulate this pathway in HCC remain poorly defined.

Recent studies have highlighted secreted proteins as critical modulators of tumor biology and promising therapeutic targets (Quail and Joyce, 2013; Zhang et al., 2013). Acting as mediators of intercellular communication, secreted proteins orchestrate signaling within the tumor microenvironment, influence metastatic behavior, and often serve as accessible biomarkers for cancer diagnosis and prognosis (Quail and Joyce, 2013). In this context, the Cell Growth Regulatory Gene 11 (CGR11) molecule has emerged as a potentially relevant factor. CGR11 encodes a secreted protein containing EF-hand calcium-binding domains and is broadly expressed in several tissues, including the liver, kidney, and small intestine (Fagerberg et al., 2014). Previous studies have shown that CGR11 regulates cell proliferation and differentiation, and its aberrant overexpression has been documented in multiple solid tumors (Díaz de la Guardia-Bolívar et al., 2022; Wei et al., 2024; Gao et al., 2025).

Functionally, CGR11 binds calcium ions and activates oncogenic pathways such as MAPK and Wnt/β-catenin, thereby promoting tumor cell proliferation and survival (Devnath et al., 2009; Deng et al., 2015; Wei et al., 2024). These findings suggest that CGR11 may act as a tumor-promoting factor, yet its biological significance and function in HCC remain largely unclear.

Through comprehensive bioinformatic analyses of public datasets (GEO, TCGA, and CPTAC) and validation in clinical HCC specimens, we identified a marked upregulation of CGR11 in HCC tissues. Increased CGR11 expression correlated with advanced clinicopathological features and poor patient prognosis. Mechanistically, our data indicate that CGR11 promotes HCC progression by inhibiting autophagy via activation of the PI3K/AKT signaling pathway. Collectively, these findings identify CGR11 as a novel regulator of the PI3K/AKT-autophagy axis and suggest that targeting CGR11 may provide a promising therapeutic strategy and prognostic biomarker for HCC.

## 2 Methods

### 2.1 Bioinformatics analysis of CGR11 expression

The CGR11 mRNA expression, clinical, and survival data for Liver Hepatocellular Carcinoma (LIHC) were obtained from The Cancer Genome Atlas (TCGA) via the UCSC Xena browser (http://xena.ucsc.edu/) (Goldman et al., 2020). Normal liver tissue RNA-seq data were obtained from the Genotype-Tissue Expression (GTEx) database. Additional validation was performed using GEO datasets (GSE36376, GSE57957, GSE76427, GSE25097, GSE36411, GSE45436, and GSE54236). Protein expression profiles from the Clinical Proteomic Tumor Analysis Consortium (CPTAC) were accessed through UALCAN (https://ualcan.path.uab.edu/) (Chandrashekar et al., 2022). GEPIA2 (http://gepia2.cancer-pku.cn/) was used to compare CGR11 expression across tumor and normal tissues, and to analyze overall (OS) and recurrence-free survival (RFS). Prognostic significance was validated using the KM Plotter database (http://www.kmplot.com).

### 2.2 Analysis of tumor microenvironmental characteristics

The expression of CGR11 in different cell types (including individual malignant cells) within LIHC was analyzed via Human Liver Browser and Single-cell Atlas in Liver Cancer (scAtlasLC) (Ma et al., 2021). CGR11 expression and immune infiltration across cancers were analyzed through the TIMER2.0 platform (Li et al., 2020), which also provided correlations between immune cell abundance and CGR11 levels.

### 2.3 Cell-cell communications analysis in scRNA-seq data

Cell-cell communication networks were inferred using the *CellChat* R package (Jin et al., 2021), based on ligand-receptor interactions in the CellChatDB database. Interaction probabilities were summarized to construct intercellular communication networks and assess signaling between CGR11-high malignant clusters and immune-related populations.

### 2.4 DNA methylation analysis

CGR11 methylation (CpG β-values) and gene expression correlations were evaluated via MethSurv (Modhukur et al., 2018) and the SMART platform (http://www.bioinfo-zs.com/smartapp/). CpG sites were classified according to genomic context, and their prognostic significance was analyzed using Kaplan-Meier plots (log-rank test, *P* < 0.05).

### 2.5 Gene enrichment analysis

Gene ontology (GO), Kyoto Encyclopedia of Genes and Genomes (KEGG) analysis and Gene Set Enrichment Analysis (GSEA) were performed using the *cluster Profiler* package. Adjusted *P* < 0.05 indicated statistical significance.

### 2.6 Immune infiltration analysis

The *GSVA* package (ssGSEA method) was used to quantify immune cell infiltration related to CGR11 expression (Hänzelmann et al., 2013). Stromal, immune, and ESTIMATE scores were calculated using the *ESTIMATE* R package, and Spearman correlations between CGR11 expression and immune infiltration were computed using the *psych* package.

### 2.7 RNA extraction and quantitative real-time PCR (RT-qPCR)

Total RNA was extracted using TRIzol™ (Invitrogen) and reverse-transcribed with the Beyotime cDNA synthesis kit. GAPDH served as the internal control. qPCR was performed using BeyoFast™ SYBR Green on a QuantStudio 3 system (Applied Biosystems). Relative expression was calculated using the 2^−ΔΔCT^ method.

Primers: GAPDH-F:5′-GAACGGGAAGCTCACTGG-3′,

GAPDH-R:5′-GCCTGCTTCACCACCTTCT-3’;

CGR11-F:5′-ACGATGACAGTGTTAATCCTGC-3′,

CGR11-R:5′-CCTAGTCCCTTTAGGTAGCTCTG-3’.

### 2.8 Cell culture and reagents

HCC cell lines PLC/PRF/5, Hep3B,and SNU449, as well as Human normal liver cells THLE2 were obtained from the American Type Culture Collection (ATCC, Rockville, MD). HCCLM3 and MHCC-97H were purchased from the Cell Bank of the Chinese Academy of Sciences (Shanghai). Cells were cultured in high-glucose DMEM with 10% fetal bovine serum (FBS, BioInd) and 1% penicillin-streptomycin (Biosharp, BL505A) at 37 °C with 5% CO_2_. MK2206 (#HY-10358) and SC79 (#HY-18749) were purchased from MedChemExpress and dissolved in DMSO at 5 μM and 4 μM, respectively.

### 2.9 Western blot analysis

Isolate total proteins from frozen HCC tissues and cells by utilizing RIPA buffer containing protease inhibitors. Then the extracted total proteins were quantified by BCA assay, separated by SDS-PAGE (NCM, Suzhou, China), and transferred to PVDF membranes. After blocking, membranes were incubated with relevant antibodies (Supplementary Table S1) and visualized using ECL chemiluminescence kit (NCM, Suzhou, China).

### 2.10 Immunohistochemistry(IHC)

IHC was performed on paraffin-embedded sections using a two-step detection kit (ZSGB-BIO, Beijing, China). Briefly, after deparaffinization, hydration, antigen retrieval, and blocking, sections were sequentially incubated with primary antibody and reaction enhancers before visualization. The IHC score of target proteins was performed based on both the staining intensity and the proportion of positively stained cells (Su et al., 2014). Briefly, staining intensity was graded as follows: 0, negative; 1, weak; 2, moderate; and 3, strong. The percentage of positive cells was scored as: 0 for <5%, 1 for 5%–25%, 2 for 26%–50%, 3 for 51%–75%, and 4 for >75%. The final immunostaining score, obtained by combining these two parameters, ranged from 0 to 12.

### 2.11 Vector construction and transfection

Overexpression and knockdown lentiviruses as well as control lentiviruses were purchased from Shanghai He Yuan Biological Co. Full-length CGR11 cDNA was cloned into lentiviral vectors for overexpression. shRNA sequences targeting CGR11 were used for knockdown. Lentivirus infection and puromycin selection (2 μg/mL, Biosharp, BL528A) generated stable lines. CGR11 expression was validated by RT-qPCR and Western blot. Transfections were performed using Lipofectamine 2000 (Invitrogen).

### 2.12 Cell proliferation experiment

Cell proliferation viability was assessed by CCK-8 and colony formation assays. Approximately 1–5 × 10^3^ cells were seeded into each well of a 96-well plate. Following cell attachment, 10 μL of CCK-8 reagent was added to each well and incubated in a cell culture incubator for 1 h. Absorbance measurements were performed at 450 nm using a microplate reader.

The colony formation assay was performed by seeding 4 × 10^2^ cells per well in 6-well plates. Cells were cultured for 2 weeks in a humidified incubator (37 °C, 5% CO_2_) to allow colony formation. Subsequently, cell colonies were fixed and stained with 1% crystal violet solution (Beyotime Biotechnology, Shanghai, China) for quantitative analysis. Colonies containing ≥50 cells were counted under bright-field microscopy to evaluate clonogenic survival capacity.

### 2.13 EdU assay

EdU assay (RiboBio, Guangzhou, China) was used to evaluate DNA synthesis. HCC cells were seeded into 24-well plates at a density of 1 × 10^5^ cells per well and incubated with 300 μL of culture medium containing 50 μM EdU for 2 h. Cells were fixed and stained according to the manufacturer’s protocol. Fluorescence imaging was performed using an inverted fluorescence microscope (Nikon Eclipse Ti2). The percentage of EdU-positive cells was quantified by ImageJ software (NIH). All experiments included triplicate biological replicates.

### 2.14 Wound healing assay and transwell invasion assay

HCC cells were inoculated in six-well plates and cultured in DMEM medium. A 10 μL sterile pipette tip was used to create a wound on the monolayer of cells, and the cells were cultured with the replacement medium. Photographs were taken at 0 h, 24 h and 48 h to record the width of the wound. In the Transwell invasion assay, approximately 4 × 10^5^ cells in 200 μL of serum-free DMEM medium (BD Biosciences, Franklin Lakes, NJ) were inoculated in the upper chamber of the Transwell inserts, and the lower chamber was added with DMEM medium containing 10% FBS, and placed into the cell culture incubator. After 24 h of incubation, cells invading the basement membrane were fixed with 4% para-formaldehyde for 10 min and stained with 0.1% crystal violet (Beyotime Biotechnology), observed under a microscope and photographed.

### 2.15 Autophagy analyses

Autophagy levels were assessed using multiple complementary approaches, including Western blotting, IHC and the mRFP-GFP-LC3 dual-fluorescence reporter assay. For Western blot analysis, cell samples were collected after the indicated treatments, and total cellular proteins were extracted. The expression levels of LC3 II and p62 were detected by immunoblotting to evaluate autophagic activity. To visualize autophagosome formation and monitor autophagic flux dynamically, cells were infected with a dual-fluorescent mRFP-GFP-LC3 lentivirus (Hanbio Biotechnology, Shanghai, China). After transfection, fluorescent puncta were observed under a confocal microscope (LEICA). In this system, yellow puncta (GFP + RFP+) represent autophagosomes, whereas red-only puncta (RFP+) indicate autolysosomes; thus, the ratio of red to yellow puncta reflects autophagic flux.

### 2.16 Flow cytometry

The proportion of apoptotic cells was detected by flow cytometry (Novocyte flow cytometer, ACEA Biosciences) using the apoptosis detection kit (Apoptosis Detection Kit, Yeasen). The data were analyzed using FlowJo (version 7.6.5).

### 2.17 Transmission electron microscopy (TEM)

After washed with pre-cooled PBS, HCC cells were fixed with electron microscopy fixative (Servicebio, Wuhan, China). Samples were dehydrated, embedded, sectioned and stained for ultrastructural observation under TEM.

### 2.18 Animal studies

BALB/c nude mice (4–5 weeks old, male), purchased from Henan Huanyu Biotechnology Co., were used for subcutaneous and orthotopic HCC xenograft models. The length (L) and width (W) of the subcutaneous tumors were measured and recorded. Tumor volume was calculated as *V* = (L × W^2^)/2. Orthotopic implantation was performed under anesthesia with pentobarbital sodium (40 mg/kg, i.p.), and tumor growth was monitored by *in vivo* fluorescence imaging system. Six weeks later, the mice were euthanized with isoflurane (5%, inhalation), and the livers and lungs were removed for pathological examination. The final volume and weight of the orthotopic tumors were measured. All animal studies were carried out in accordance with relevant laws and regulations and approved by the Institutional Animal Ethics Committee of Hunan Provincial People’s Hospital/Hunan Normal University First Affiliated Hospital (Ethics Review Number: [2025]-115).

### 2.19 RNA sequencing and data analysis

Total RNA was extracted from MHCC-97H-Vector and MHCC-97H-shCGR11 cells using Trizol reagent (Invitrogen, Carlsbad, CA). The quantity and integrity of RNA were detected by using the K5500 ultrafine spectrophotometer (Kaiao, Beijing, China) and Agilent 2200 tapstation (Agilent Technologies, Santa Clara, CA). RNA-seq data were analyzed by GSEA using Hallmark gene sets.

### 2.20 Statistical analyses

SPSS 22.0 (SPSS Inc.,Chicago, IL) and GraphPad Prism (Version 10) were used for statistical analyses. Data were shown the mean ± SD of at least three independent experiments. Student’s t tests, and Spearman and Pearson correlation were used to analyze the correlation. Univariate and multivariate analyses were conducted by the Cox proportional hazards model to identify independent risk factors. P < 0.05 is statistically significant for the difference.

## 3 Results

### 3.1 CGR11 is upregulated in multiple human tumors and HCC tissues

**FIGURE 1:**
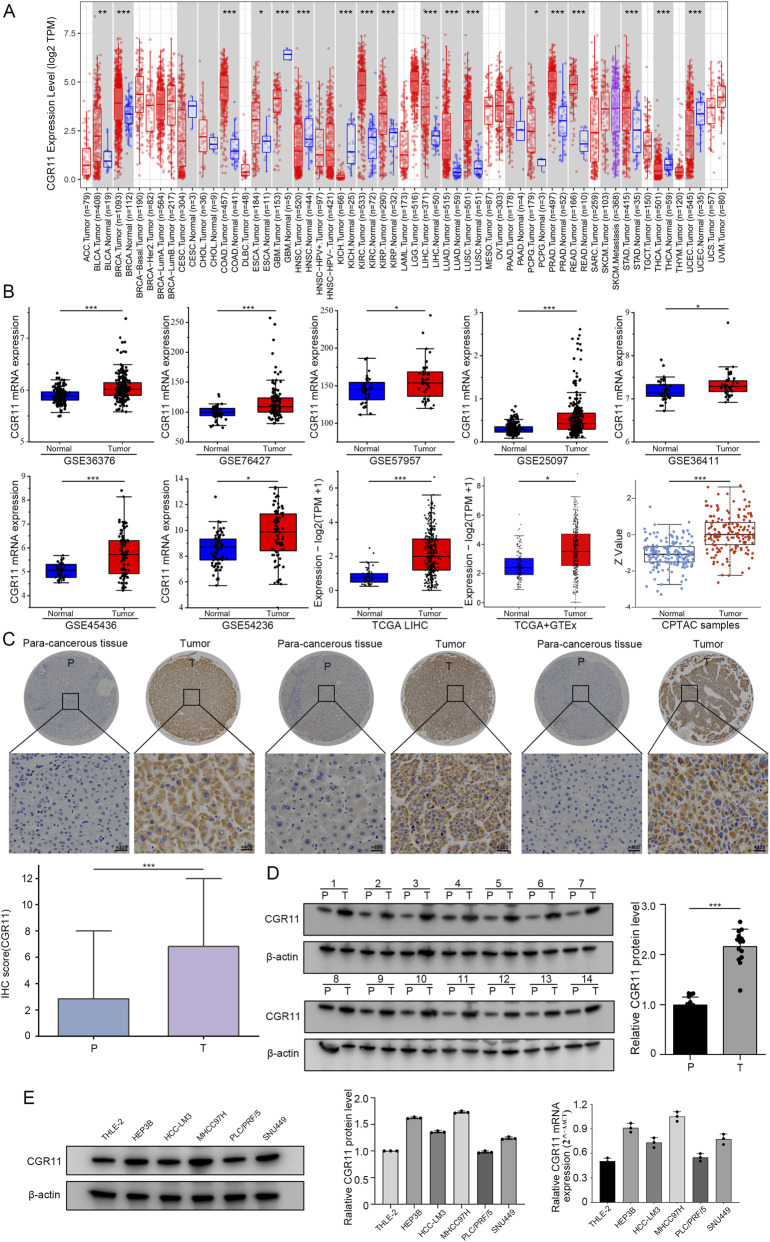
CGR11 expression is upregulated in HCC. **(A)** The expression of the CGR11 in pan-cancer analysis across various human tumors. **(B)** CGR11 expression in HCC was analyzed in publicly available databases, including CGR11 mRNA expression in GEO (GSE36376, GSE76427, GSE57957, GSE25097, GSE36411, GSE45436, and GSE54236), TCGA LIHC, GTEx and CGR11 protein expression in CPTAC databases. **(C)** Representative IHC images and IHC score of CGR11 protein expression in tissue microarray (TMA) of 100 pairs of HCC tissues (T) and paired para-cancerous tissues (P), T: tumor, P: para-cancerous tissues. **(D)** Western blot of 14 pairs of randomly selected HCC tumor and para-cancerous tissues. **(E)** Western blot and qRT-PCR were employed to evaluate CGR11 protein and mRNA expression in HCC cell lines. Data are mean ± SD of three independent experiments. *P < 0.05; **P < 0.01; ***P < 0.001.

Recent studies have shown that CGR11 is associated with the malignant characteristics of several tumors (Wei et al., 2024; Gao et al., 2025). To explore CGR11 expression across cancers, we analyzed mRNA and protein levels using TIMER 2.0 and Xiantao Academic databases. CGR11 showed high expression in bladder, breast, colon, kidney, esophageal, liver, lung, prostate, rectal, and gastric cancers, while showing low expression in glioblastoma, head and neck, thyroid, and uterine cancers (Figure 1A). In TCGA, GTEx, GEO, and CPTAC datasets, CGR11 expression was markedly increased in HCC compared to normal liver tissues (Figure 1B). Consistent results were obtained from multiple GEO datasets (GSE36376, GSE76427, GSE57957, GSE25097, GSE36411, GSE45436, and GSE54236). CPTAC data confirmed that CGR11 protein levels were significantly higher in HCC tissues.

IHC analysis of TMA including 100 paired clinical specimens demonstrated increased CGR11 staining in tumor tissues relative to adjacent non-tumor liver (Figure 1C). Similarly, Western blotting and qRT-PCR of 14 paired tissues verified higher CGR11 expression in HCC (Figure 1D; Supplementary Figure S1). The expression of CGR11 was also found to be increased in liver cancer cell lines (Hep3B, HCC-LM3, MHCC-97H, SNU449) compared to primary hepatocytes (THLE-2), while no significant difference was observed in PLC/PRF/5 (Figure 1E). The results of qRT-PCR analysis were consistent with those of Western blot, further validating the expression changes of CGR11 in liver cancer cell lines (Figure 1E). Collectively, these results reveal that CGR11 is significantly overexpressed in HCC.

### 3.2 Association between CGR11 expression and the tumor microenvironment (TME)

**FIGURE 2:**
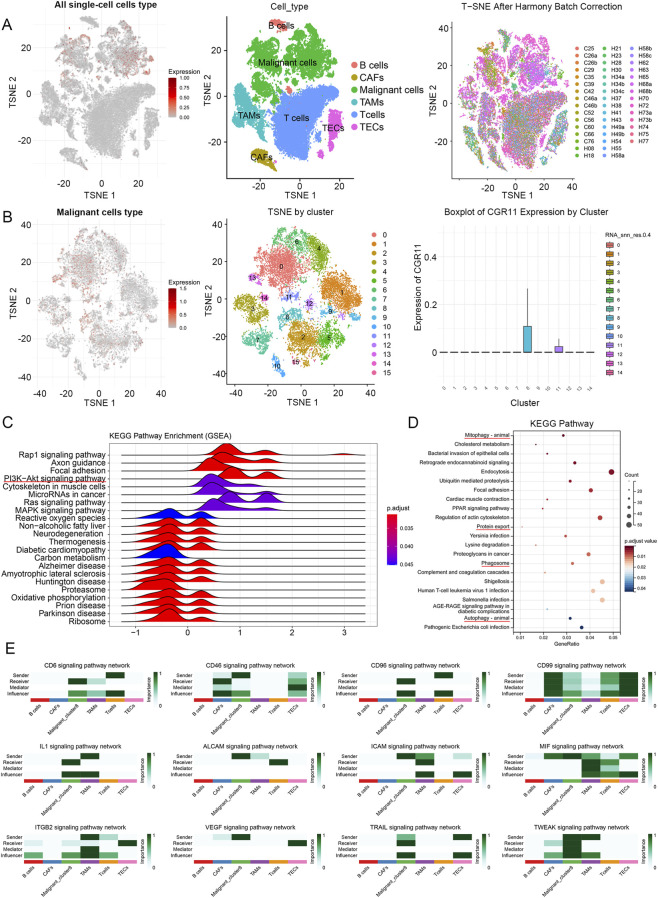
Characterization of CGR11 expression on TME in the public datasets (the scAtlasLC dataset and CellChatDB database). **(A)** Single-cell CGR11 gene expression analysis in all single-cell cells type of HCC using scAtlasLC. **(B)** Single-cell CGR11 gene expression analysis and malignant cells clustering analysis were performed following malignant cells type extraction. **(C)** GSEA and **(D)** KEGG pathway enrichment analysis were performed between CGR11-high clusters (Malignant_cluster8) and other CGR11-low clusters within malignant cells type. **(E)** Cell-cell communication analysis between the cluster Malignant_cluster8 with the highest expression of CGR11 and various immune-related molecules.

To characterize the cellular distribution of CGR11, we analyzed the scAtlasLC single-cell dataset. CGR11 was predominantly expressed in malignant hepatocytes, with low expression in immune and stromal cells, including T cells, B cells, CAFs, TAMs, and TECs (Figure 2A). Clustering analysis identified 15 malignant subclusters, with cluster 8 showing the highest CGR11 expression (Figure 2B). GSEA and KEGG analyses revealed enrichment of pathways related to PI3K/AKT, MAPK signaling, autophagy, oxidative phosphorylation, and protein export in the CGR11-high cluster (Figures 2C,D). Cell-cell interaction analysis demonstrated strong communication between CGR11-high tumor cells and immune components (TAMs, TECs, T cells) via ligand-receptor pairs such as CD6, CD46, MIF, VEGF, and TRAIL (Figure 2E). DNA methylation analysis (SMART and MethSurv databases) indicated reduced CGR11 methylation in HCC tissues, with low CpG methylation levels correlating with poorer survival prognosis (Supplementary Figure S3). These findings suggest that CGR11 upregulation in HCC may be partly due to hypomethylation and is associated with immune-related signaling within the TME.

### 3.3 High CGR11 expression predicts poor prognosis in HCC

**FIGURE 3:**
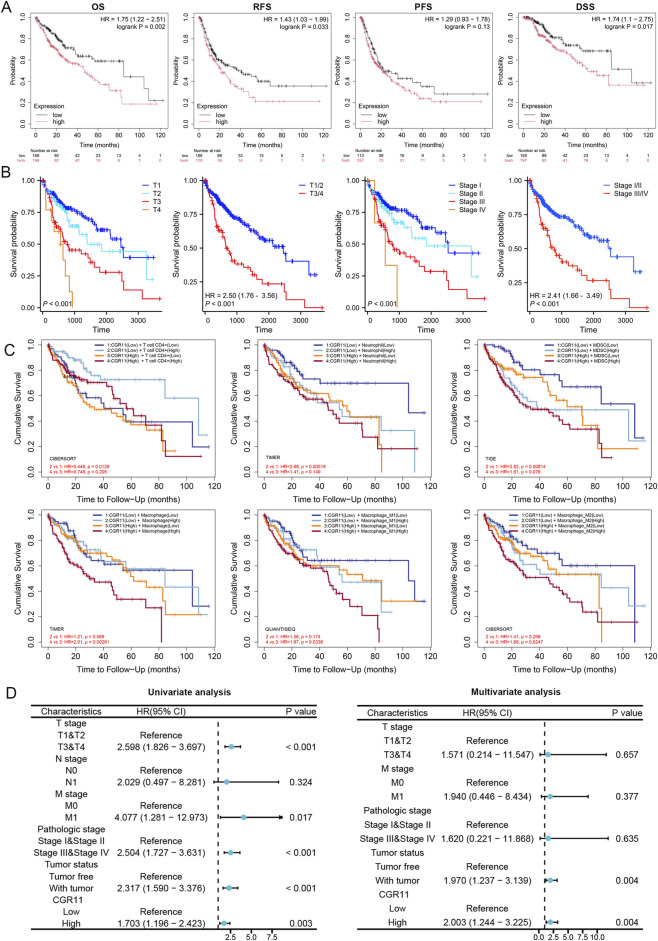
The prognostic value of CGR11 in HCC. **(A)** The prognostic value of CGR11 expression stratified by Kaplan-Meier survival analysis: overall survival (OS), relapse-free survival (RFS), progression-free survival (PFS), and disease-specific survival (DSS). **(B)** Overall survival analysis of CGR11 expression stratified by tumor grade and clinical stage. **(C)** Comprehensive prognostic value of CGR11 expression and T cell CD4^+^, macrophage, macrophage M1, macrophage M2, Neutrophil, and MDSC infiltration levels based on the TIMER algorithm. **(D)** Forest plot of CGR11-associated overall survival hazard ratio (TCGA-LIHC cohort, univariate analysis and multivariate analysis). HR, hazard ratio.

Kaplan-Meier analysis using the TCGA-LIHC cohort revealed that high CGR11 expression correlated with shorter overall survival (OS), relapse-free survival (RFS), and disease-specific survival (DSS), though not with progression-free survival (PFS) (Figure 3A). Stratification by clinical stage and tumor grade showed that patients with low tumor grade or early-stage disease had longer survival (Figure 3B). The clinical characteristics of HCC patients are listed in Supplementary Table S2. Immune infiltration analysis indicated that high CGR11 expression combined with increased CD4^+^ T cells, macrophages, neutrophils, and MDSCs predicted worse prognosis (Figure 3C). No significant association was found for CD8^+^ T or B cells. CIBERSORTx analysis and GEO meta-analysis were also explored CGR11-immune interaction (Supplementary Figure S4). Univariate Cox analysis identified T stage, M stage, overall clinical stage, and CGR11 expression as risk factors for OS. Multivariate analysis confirmed CGR11 as an independent prognostic biomarker for poor survival (Figure 3D).

These results collectively demonstrated that high CGR11 expression was correlated with poor prognosis of HCC patients and could serve as a valuable independent prognostic factor.

### 3.4 CGR11 promotes proliferation, migration, invasion, and inhibits apoptosis *in vitro*

**FIGURE 4:**
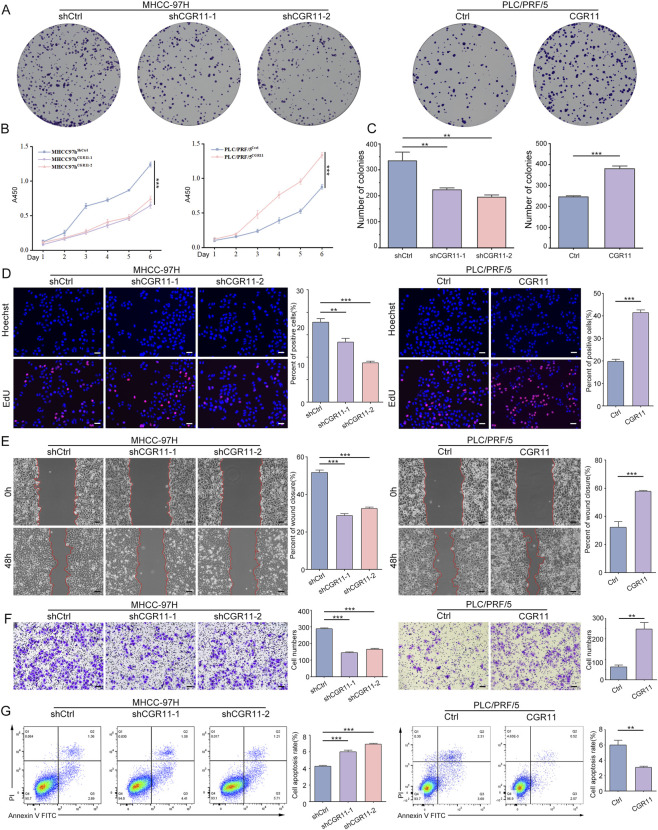
CGR11 inhibits proliferation, migration, invasion and inhibits apoptosis of HCC cells *in vitro*. **(A)** Clonogenic capacity of HCC cell lines with CGR11 overexpression or knockdown were assessed by colony formation assay. **(B)** CCK8 assay showed the proliferative curves of CGR11-knockdown MHCC-97H, CGR11-overexpressing PLC/PRF/5 and their control cells. **(C)** Statistical chart of MHCC-97H and PLC/PRF/5 cells in colony formation assay. **(D)** EdU assays showed the proliferating cells in MHCC-97H^shCGR11^, PLC/PRF/5^CGR11^ and their control groups. Nuclei of proliferating cells were labelled by EdU (red), and all nuclei of HCC cells were labelled by Hoechst 33,342 (blue). The percentage of proliferating cells was compared in corresponding bar chart. Scale bars, 50 μm. **(E)** Wound healing assay showed the migration ability of MHCC-97H^shCGR11-1^, MHCC-97H^shCGR11-2^, PLC/PRF/5^CGR11^ and their control cells at 0 and 48 h. Scale bars, 50 μm. **(F)** Transwell invasion assay showed the invasion ability of MHCC-97H^shCGR11-1^, MHCC-97H^shCGR11-2^, PLC/PRF/5^CGR11^ and their control cells at 24 h. Scale bars, 50 μm. **(G)** The rate of apoptosis in HCC cells with CGR11 knockdown or overexpression and in their control cells was analyzed by flow cytometry. Data shown as mean ± SD of triplicate independent experiments. **P < 0.01; ***P < 0.001.

Based on endogenous expression, MHCC-97H cells were used for CGR11 knockdown, and PLC/PRF/5 cells for overexpression (validation shown in Supplementary Figures S1,S2). Colony formation assays, CCK-8 proliferation assays and EdU incorporation experiments demonstrated that knockdown of CGR11 significantly inhibited cell proliferation and colony formation, whereas its overexpression promoted cell growth (Figures 4A–D). Wound-healing and Transwell assays showed that CGR11 knockdown significantly reduced cell migration and invasion, whereas CGR11 overexpression had the opposite effect (Figures 4E,F). Flow cytometry revealed that CGR11 knockdown increased apoptosis, while CGR11 overexpression suppressed it (Figure 4G). Taken together, these results suggest that CGR11 enhances the malignant behavior of HCC cells *in vitro*.

### 3.5 CGR11 promotes HCC tumor growth *in vivo*

**FIGURE 5:**
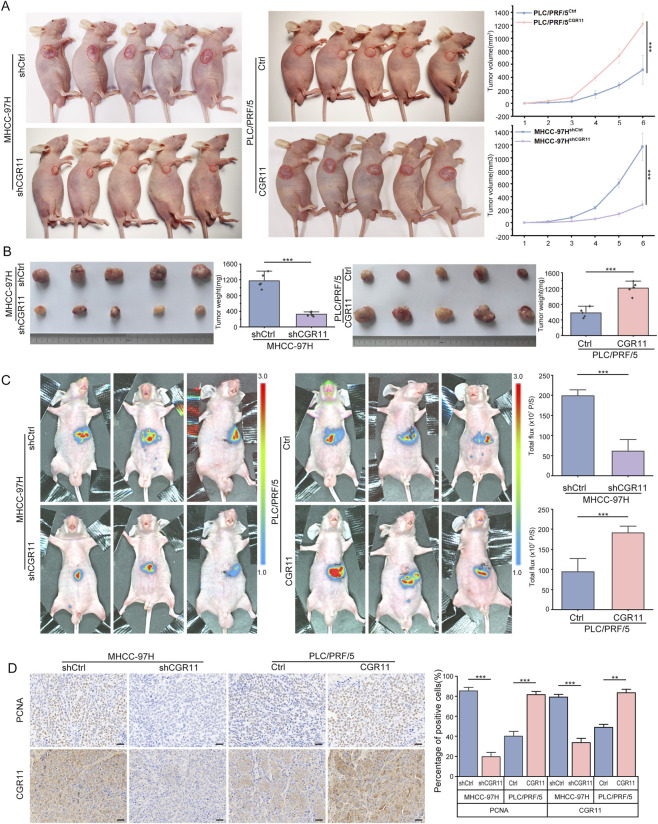
CGR11 promotes tumor growth and invasive potential of HCC cells *in vivo*. **(A)** Subcutaneous tumor model was established using MHCC-97H^shCGR11^, PLC/PRF/5^CGR11^ and the control cells (n = 5). Tumor weight and growth kinetics of subcutaneous xenografts were quantified and comparatively analyzed. **(B)** Subcutaneous tumors removed from nude mice of MHCC-97H^shCGR11^, PLC/PRF/5^CGR11^ and the control cells (n = 5) were harvested and photographed. **(C)** Orthotopic tumor models in nude mice were established using MHCC-97H^shCGR11^, PLC/PRF/5^CGR11^ and their control cells. Orthotopic liver tumors were longitudinally monitored via small-animal *in vivo* fluorescence imaging. Luciferase activity of orthotopic tumors were compared in the left panel. **(D)** PCNA and CGR11 expression levels in subcutaneous tumor models derived from MHCC-97H^shCGR11^, PLC/PRF/5^CGR11^ and their control cells were detected by IHC, respectively. Scale bars, 200 μm **P < 0.01; ***P < 0.001.

To further investigate the role of CGR11 *in vivo*, we established subcutaneous xenograft and orthotopic implantation models in nude mice. We divided the experimental cohorts into four groups: MHCC-97H-Control, MHCC-97H-shCGR11, PLC/PRF/5-Control, and PLC/PRF/5-CGR11-Overexpression. CGR11 knockdown significantly reduced tumor size and weight, while CGR11 overexpression markedly enhanced tumor growth (Figures 5A,B). The orthotopic tumor model further confirmed that CGR11 knockdown suppressed intrahepatic tumor growth by ∼72%, whereas CGR11 overexpression accelerated progression by 2.3-fold (Figure 5C). Subsequent IHC staining of PCNA expression further supported that CGR11 enhanced proliferative activity *in vivo* (Figure 5D). Collectively, these findings confirm that CGR11 promotes HCC tumor growth.

### 3.6 CGR11 influences the PI3K/AKT pathway in HCC cells

**FIGURE 6:**
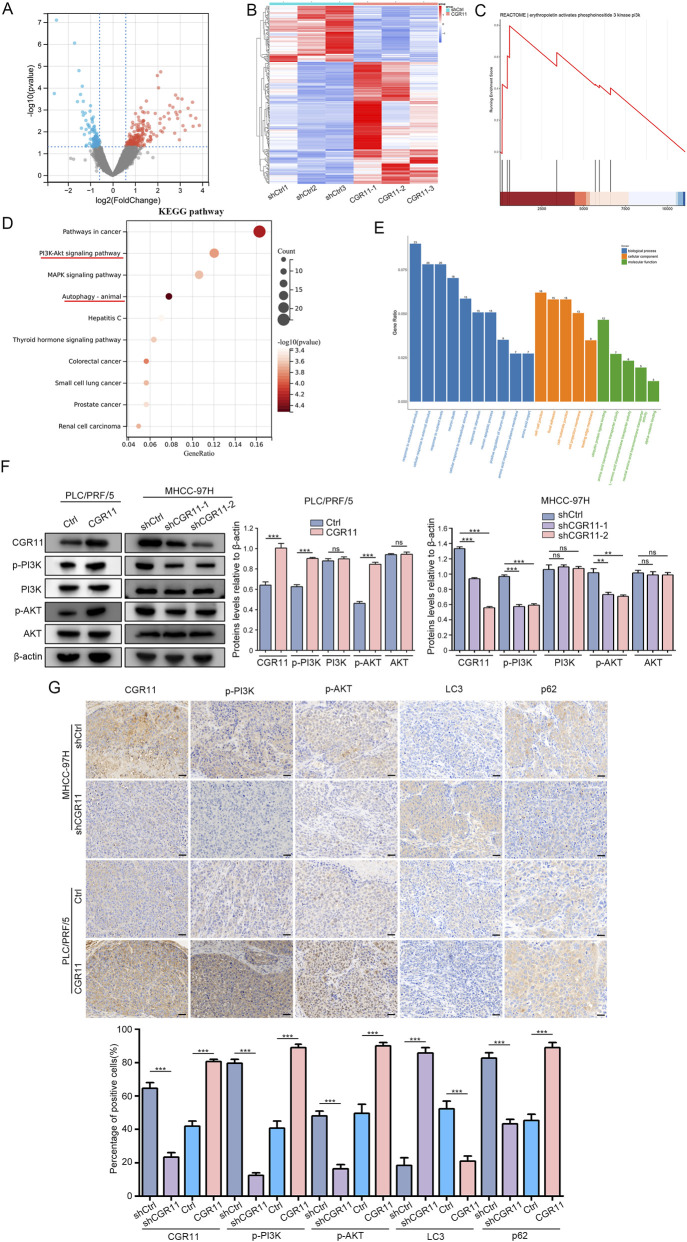
CGR11 influences the PI3K/AKT pathway in HCC cells. **(A)** RNA-seq analysis of the differential expression genes of MHCC-97H^shCGR11^ cells and the control cells. The volcano diagram shows that differential genes expression after knockdown of CGR11. **(B)** Heat-map shows these common altered differential genes expression in MHCC-97H^shCGR11^ cells and the control cells. **(C)** Representative results of PI3K-related pathways from Gene Set Enrichment Analysis (GSEA) of CGR11 in RNA-seq analysis of MHCC-97H^shCGR11^ cells and the control cells. **(D)** KEGG enrichment analysis for significantly differential expression genes from RNA-seq analysis. **(E)** Gene Ontology (GO) enrichment analysis of significantly differentially expressed genes (DEGs) between CGR11 knockdown and control groups in RNA-seq profiling. **(F)** Western blot analysis and statistical analysis of CGR11, p-PI3K, p-AKT and their total protein expression levels in MHCC-97H^shCGR11^, PLC/PRF/5^CGR11^ and their control cells. **(G)** IHC images and statistical analysis of CGR11, p-PI3K, p-AKT, LC3 and p62 protein expression in subcutaneous tumors removed from nude mice. Scale bars, 100 μm. The data are presented as mean ± SD of three independent experiments. *P < 0.05; **P < 0.01; ***P < 0.001.

To further elucidate the underlying mechanisms, RNA-seq analysis of MHCC-97H-shCGR11 cells identified 282 differentially expressed genes through Volcano plot analysis (Figure 6A). A hierarchical clustering heatmap demonstrated clear differences in transcriptional profiles between MHCC-97H shCGR11 cells and the control group (Figure 6B). KEGG and GSEA results highlighted significant involvement of PI3K/AKT, MAPK, and autophagy pathways (Figures 6C,D). GO Enrichment analysis revealed that CGR11 regulates pathways related to extracellular stimulus response, cell-matrix interaction, and transmembrane transport, predominantly localized at the membrane (Figure 6E). Western blot validation confirmed that CGR11 knockdown decreased the levels of phosphorylated PI3K(p-PI3K) and AKT (p-AKT), while CGR11 overexpression increased the levels of p-PI3K and p-AKT (Figure 6F). IHC of xenografts showed a positive correlation among CGR11, p-PI3K, p-AKT, LC3 and p62 expression (Figure 6G), confirming that CGR11 activates the PI3K/AKT pathway. These findings align with our prior single-cell transcriptomic profiling results (Figure 2G).

### 3.7 CGR11 influences autophagy in HCC cells

**FIGURE 7:**
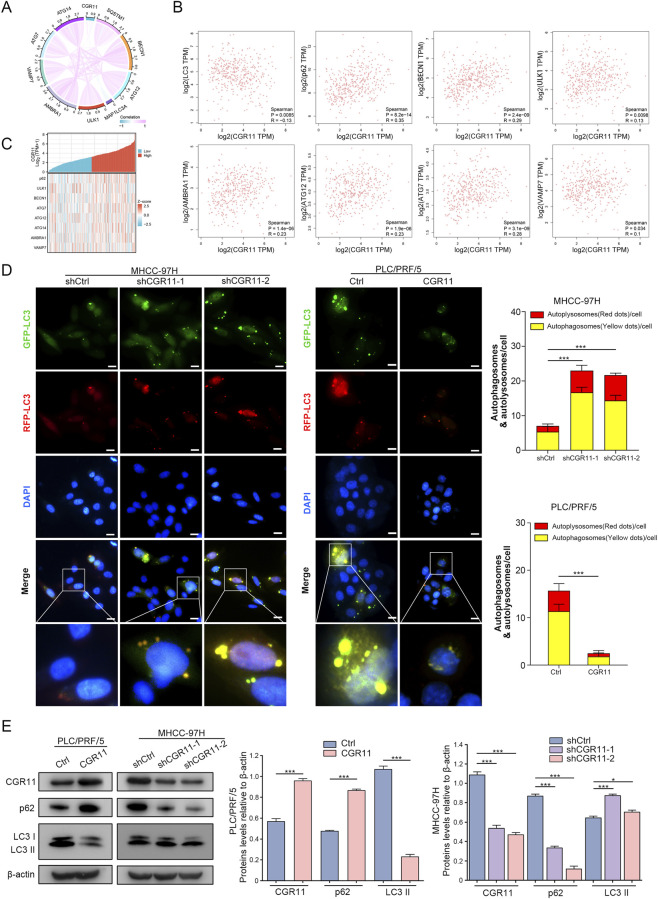
CGR11 influences autophagy in HCC cells. **(A)** Correlation chord diagram depicting the association network between CGR11 and autophagy-related proteins based on expression profiling. **(B)** Correlation scatter plots between CGR11 and autophagy-associated proteins with annotated Pearson coefficients (r) and significance levels (p-values). **(C)** A co-expression heat-map delineates the correlation patterns between CGR11 and autophagy-associated proteins, with hierarchical clustering revealing expression synergy across biological replicates. **(D)** Fluorescence images and statistical analysis of MHCC-97H^shCGR11^, PLC/PRF/5^CGR11^ and the corresponding control cells after transfecting with mRFP-GFP-LC3 lentivirus. Red dots represent autolysosomes while yellow dots indicate autophagosomes in the overlays. Nuclei were stained with DAPI. The average number of autophagosomes and autolysosomes per cell was quantified. A total of 50 cells from randomly selected fields in each group were counted for the analysis. Scale bars, 5 μm. **(E)** Western blot analysis and statistical analysis of CGR11, LC3 II and p62 levels in HCC cells with CGR11 overexpression or knockdown. Data shown as mean ± SD of triplicate independent experiments. *P < 0.05; **P < 0.01; ***P < 0.001.

Given CGR11’s functional association with autophagy-related pathways, we hypothesized its regulatory role in modulating autophagic processes in HCC. Public database analyses showed a negative correlation between CGR11 expression and autophagy-related markers (LC3, p62/SQSTM1) (Figures 7A–C). IHC analysis of LC3 and p62 in xenografts from mice with CGR11 overexpression and downregulation models revealed a negative correlation between LC3II expression and CGR11 levels, whereas p62 expression showed a positive correlation with CGR11 (Figure 6G). Using the mRFP-GFP-LC3 reporter assay, we observed that CGR11 overexpression decreased autophagic flux in HCC cells, whereas knockdown enhanced autophagic flux (Figure 7D). As p62 is delivered to autolysosomes via membrane binding for LC3II degradation, cytoplasmic p62 accumulation indicates impaired autophagic degradation. Western blot analysis further confirmed that CGR11 knockdown increased LC3-II levels and decreased p62 levels, while overexpression showed the opposite effects (Figure 7E). TEM revealed abundant autophagosomes in CGR11-knockdown cells, but markedly fewer in CGR11-overexpressing cells (Supplementary Figure S5A). Taken together, these data indicate that CGR11 inhibits autophagy, likely through PI3K/AKT pathway activation.

### 3.8 CGR11 inhibits autophagy by regulating the PI3K/AKT pathway

**FIGURE 8:**
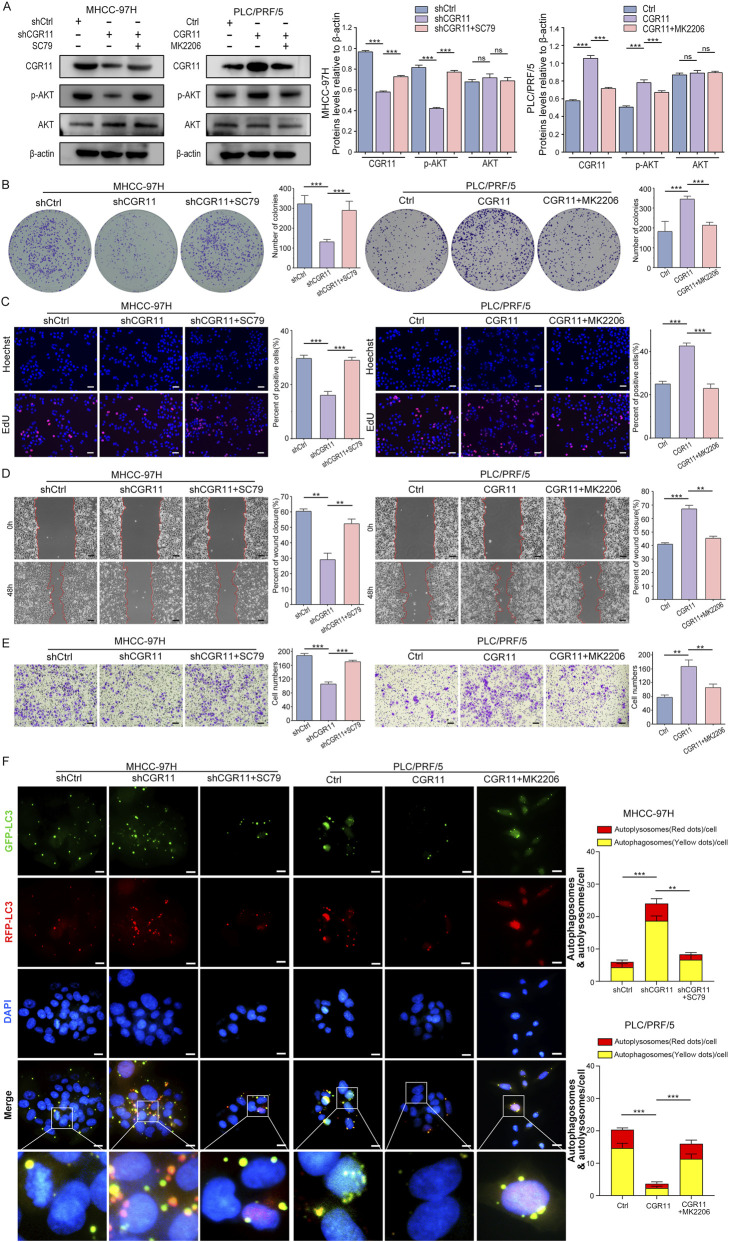
CGR11 regulates PI3K/AKT-mediated autophagy to promote HCC cell progression. **(A)** Western blot analysis of CGR11, p-AKT, and AKT expression in HCC cells with CGR11 overexpression or knockdown after further treatment with SC79 (4 μM) or MK2206 (5 μM). **(B,C)** Colony formation assays and EdU assays showed the proliferation ability of the indicated HCC cells after further treatment with SC79 (4 μM) or MK2206 (5 μM). **(D,E)** After treatment with SC79 (4 μM) or MK2206 (5 μM), the migration and invasion ability of HCC cells with knockdown or overexpression of CGR11 was determined by wound healing and Transwell assays, respectively. Scale bars, 50 μm. **(F)** Fluorescence images of MHCC-97H^shCGR11^, PLC/PRF/5^CGR11^ and the corresponding control cells, transduced with different mRFP-GFP-LC3 lentivirus, after treatment with SC79 (4 μM) or MK2206 (5 μM). Red dots represent autolysosomes while yellow dots indicate autophagosomes in the overlays. Nuclei were stained with DAPI. The average number of autophagosomes and autolysosomes per cell was quantified. A total of 50 cells from randomly selected fields in each group were counted for the analysis. Data shown as mean ± SD of triplicate independent experiments. *P < 0.05; **P < 0.01; ***P < 0.001.

Given that PI3K/AKT suppresses autophagy through ULK1/2 inhibition and PTEN interaction, we investigated whether CGR11 acts through this axis (Wang et al., 2018; Quan et al., 2025). Treatment with the AKT activator (SC79) restored p-AKT levels and cell proliferation in CGR11-knockdown cells, whereas the AKT inhibitor (MK2206) reversed the tumor-promoting effects of CGR11 overexpression (Figures 8A–C). Similarly, wound healing and invasion assays showed that modulation of AKT reversed CGR11-mediated effects on cell migration and invasion (Figures 8D,E). LC3 dual-fluorescence reporter assays demonstrated that SC79 suppressed autophagy in CGR11-knockdown cells, while MK2206 restored autophagy in CGR11-overexpressing cells (Figure 8F). TEM results also showed that treatment with SC79 reduced the numbers of autophagosomes, whereas treatment with MK2206 increased their numbers (Supplementary Figure S5B). Collectively, these findings indicate that CGR11 promotes HCC proliferation and invasion by activating PI3K/AKT signaling to inhibit autophagy.

## 4 Discussion

Current research on the molecular mechanisms underlying the malignant progression of HCC remains limited, and the intricate interplay between oncogenic drivers and epigenetic regulatory networks in mediating tumor plasticity yet to be fully elucidated (Wu et al., 2020). CGR11, a classical pathway-regulated secretory protein, has demonstrated prognostic significance in prostate cancer and osteosarcoma (Díaz de la Guardia-Bolívar et al., 2022; Wei et al., 2024). However, the precise molecular mechanisms by which CGR11 contributes to HCC progression remain obscure. A recent study by Gao et al. (Gao et al., 2025) identified CGR11 as a regulatory factor promoting HCC cell proliferation and migration via the EIF3H-mediated Wnt/β-catenin signaling axis. Nevertheless, that study lacked comprehensive *in vivo* validation, as well as sufficient *in vitro* and genomic analyses such as RNA sequencing to, substantiate the proposed EIF3H-Wnt/β-catenin pathway.

In contrast, our study integrated bioinformatics analyses, molecular assays, RNA sequencing, and functional validations in both *in vitro* and *in vivo* models including subcutaneous and orthotopic xenografts, to define how CGR11 facilitates HCC malignant progression through activation of the PI3K/AKT pathway and suppression of autophagy. Through integrated multi-omics analysis of TCGA, GEO, and CPTAC datasets, we demonstrated that CGR11 is significantly upregulated in HCC tissues, correlates positively with aggressive clinicopathological features, and serves as an independent prognostic factor for both OS and RFS. Furthermore, CGR11 overexpression was significantly associated with aberrant DNA methylation and immune cell infiltration within the tumor microenvironment. In conjunction with single-cell transcriptomic data showing predominant CGR11 expression within malignant cell clusters, these findings suggest that CGR11 may not only function as a critical regulator promoting HCC progression but also as a participant in intercellular communication and immune modulation within the TME. Recent study has emphasized extracellular vesicles (EVs) as vital mediators of metabolic and signaling crosstalk between tumor and stromal compartments (Encarnação CC et al., 2024). It is plausible that CGR11 may influence or interact with EV-associated metabolic and immune communication networks. Future investigations employing EV profiling, co-culture systems, or spatial-omics approaches could further elucidate how CGR11-driven PI3K/AKT activation integrates with TME remodeling and metabolic adaptation.

Mechanistically, our functional studies revealed that CGR11 sustains malignant phenotypes in HCC cells by inhibiting autophagy levels through activation of the PI3K/AKT pathway. Consistent with this, single-cell transcriptomic profiling confirmed predominant CGR11 overexpression specifically within malignant hepatocyte clusters. Functionally, CGR11 overexpression enhanced HCC cell proliferation, invasion, and migration, whereas its knockdown markedly attenuated these phenotypes. Comprehensive *in vitro* and *in vivo* assays confirmed CGR11’s oncogenic role in maintaining HCC malignancy and promoting tumor growth.

Integrated RNA-seq and single-cell analyses further identified the PI3K/AKT signaling axis as a major pathway downstream of CGR11. The PI3K/AKT pathway is a well-established regulator of proliferation, survival, and metabolism, and its aberrant activation in HCC has been consistently linked to tumor progression, metastasis, and drug resistance (Li et al., 2022; Wan et al., 2024). This process, initiated by PI3K-mediated activation of AKT and subsequent phosphorylation of targets such as mTOR, promotes cell growth. Another study, including one identifying RNA editing as a driver of hepatocarcinogenesis via the COPA-PI3K/AKT axis (Song et al., 2021), further underscores the centrality of this pathway in HCC biology.

Autophagy is a lysosome-dependent degradation mechanism that maintains cellular homeostasis by eliminating damaged organelles and macromolecules, playing a dual role in HCC. While it can suppress malignant transformation at early stages by removing abnormal proteins and organelles, it often supports tumor survival and therapeutic resistance in advanced disease by providing metabolic flexibility (Debnath et al., 2023). Therefore, it is crucial to dissect the dynamic regulatory networks that govern autophagy in order to develop targeted HCC therapy.

Furthermore, insights from traditional medicine and ion channel research provide additional translational relevance. A paradigm shift is underway in cancer therapy, with novel strategies that concurrently target oncogenic signaling pathways and modulate the TME, ultimately reshaping the treatment landscape (Joshi et al., 2024). Liu et al. have demonstrated that certain toxic components of traditional Chinese medicine exert potent anti-cancer effects through modulation of ion channels and autophagy pathways (Liu, 2021; 2022; 2023). These findings underscore the intricate convergence between autophagy regulation, ion flux, and therapeutic response. Moreover, the intricate crosstalk between PI3K/AKT signaling and autophagy represents a pivotal axis in cancer progression. PI3K/AKT activation has been shown to promote tumor-enhancing autophagy (Li et al., 2024) and to facilitate oncogenesis by repressing or functionally impairing key autophagy mediators such as Beclin-1, LC3, and p62 (Cocco et al., 2022; Wang et al., 2023). For instance, SDC1 overexpression in HCC suppresses autophagy via PI3K/AKT/mTOR activation, thereby enhancing tumor survival and metastatic potential (Yu et al., 2020). Similarly, FDX1 downregulation promotes tumor progression through coordinated activation of mitophagy and PI3K/AKT signaling linked to excessive ROS production in HCC(Sun et al., 2024). Meanwhile, NLRP6 was reported by the Sun Yat-sen University group to potentiate PI3K/AKT signaling through autophagic degradation of p85α, an effect reversed by pharmacologic inhibition (Zhi et al., 2023).

In our study, Western blotting, IHC analyses, and mRFP-GFP-LC3 flux assays revealed that CGR11-mediated PI3K/AKT activation correlates with altered expression of LC3 II and p62, indicative of autophagy inhibition. Impaired autophagy reduced the cellular capacity to remove damaged organelles and misfolded proteins, thereby enhancing malignant potential. Pharmacological experiments further confirmed that inhibition of AKT with MK2206 partially reversed CGR11-induced motility and invasion, whereas activation of AKT using SC79 exacerbated these effects. These results validate PI3K/AKT signaling as a critical downstream effector of CGR11 and are consistent with previous findings showing that SOCS5 promotes HCC metastasis through PI3K/AKT/mTOR-mediated inhibition of autophagy (Zhang et al., 2019). Collectively, our data establish CGR11 as a pivotal regulator of HCC progression by suppressing autophagy via activation of the PI3K/AKT pathway, thereby enhancing cellular proliferation and invasiveness.

Finally, *in vivo* validation using BALB/c nude mouse models confirmed that CGR11 overexpression significantly accelerated tumor growth, as evidenced by increased xenograft volume and mass, while CGR11 knockdown markedly reduced both parameters. Orthotopic liver models yielded consistent results, reinforcing the conclusion that CGR11 promotes HCC progression via PI3K/AKT pathway activation coupled with autophagy inhibition. Looking forward, in the post-pandemic era of accelerated biomedical integration, multi-omics and personalized therapeutic frameworks are becoming central to precision oncology (Chen, 2024). Positioning CGR11 within such integrative, multi-dimensional paradigms of HCC biology may facilitate its translation into novel diagnostic and therapeutic strategies.

Despite these advances, several limitations must be acknowledged. The mechanisms driving CGR11 overexpression, whether transcriptional, epigenetic, or EV-mediated, remain unclear. The link between CGR11-induced PI3K/AKT activation and tumor microenvironment remodeling also requires further study. Clarifying these processes will aid in understanding how CGR11 regulates metabolic reprogramming and therapy response, facilitating the development of targeted HCC treatments.

## 5 Conclusion

In summary, our findings identify CGR11 acts as a critical regulator promoting HCC progression through PI3K/AKT-mediated suppression of autophagy, highlighting its potential as both a prognostic biomarker and a therapeutic target.

## Data Availability

The original contributions presented in the study are included in the article/Supplementary Material, further inquiries can be directed to the corresponding authors.
