# Supplementary material for: CGR11 promotes hepatocellular carcinoma progression by regulating autophagy through the PI3K/AKT pathway
Source: Front Cell Dev Biol. 2026 Jan 7;13:1692480. doi: 10.3389/fcell.2025.1692480 (PMC12819743; doi:10.3389/fcell.2025.1692480)
Supplement: Supplementary file 1 [file Table1.docx]

**Supplementary Tables**

Table S1. The primary antibodies used in this study

| **Primary Antibodies** | **Origin** | **Application** | **Dilution** |
| --- | --- | --- | --- |
| CGR11 | Origene (TA315090) | WB | 1:1000 |
|  |  | IHC | 1:100 |
| β-actin | Proteintech (66009-1-Ig) | WB | 1:5000 |
| LC3 | Proteintech (18725-1-AP) | WB | 1:1000 |
|  |  | IHC | 1:100 |
| P62/SQSTM1 | Proteintech (66184-1-Ig) | WB | 1:2000 |
| PI3K | Cell Signaling Technology (#4249) | WB | 1:1000 |
| p-PI3K | MedChemExpress (#HY-P81211) | WB | 1:1000 |
|  |  | IHC | 1:100 |
| AKT | Cell Signaling Technology (#9272) | WB | 1:1000 |
| p-AKT | Cell Signaling Technology (#4060) | WB | 1:2000 |
|  |  | IHC | 1:100 |
| PCNA | Cell Signaling Technology (#2586) | IHC | 1:8000 |

Table S2. Clinical Characteristics of HCC Patients in TCGA database

| Characteristics | Low expression of CGR11 | High expression of CGR11 | P value |
| --- | --- | --- | --- |
| n | 187 | 187 |  |
| Pathologic T stage, n (%) |  |  | 0.077 |
| T1&T2 | 146 (39.4%) | 132 (35.6%) |  |
| T3&T4 | 39 (10.5%) | 54 (14.6%) |  |
| Pathologic N stage, n (%) |  |  | 0.716 |
| N0 | 119 (46.1%) | 135 (52.3%) |  |
| N1 | 1 (0.4%) | 3 (1.2%) |  |
| Pathologic M stage, n (%) |  |  | 1.000 |
| M0 | 124 (45.6%) | 144 (52.9%) |  |
| M1 | 2 (0.7%) | 2 (0.7%) |  |
| Histological type, n (%) |  |  | 0.521 |
| Hepatocholangiocarcinoma (mixed)&Fibrolamellar carcinoma | 4 (1.1%) | 6 (1.6%) |  |
| Hepatocellular carcinoma | 183 (48.9%) | 181 (48.4%) |  |
| Tumor status, n (%) |  |  | 0.081 |
| Tumor free | 110 (31%) | 92 (25.9%) |  |
| With tumor | 69 (19.4%) | 84 (23.7%) |  |
| Histologic grade, n (%) |  |  | 0.016 |
| G1&G2 | 128 (34.7%) | 105 (28.5%) |  |
| G3&G4 | 57 (15.4%) | 79 (21.4%) |  |
| AFP(ng/ml), n (%) |  |  | 0.109 |
| <= 400 | 117 (41.8%) | 98 (35%) |  |
| > 400 | 28 (10%) | 37 (13.2%) |  |
| Vascular invasion, n (%) |  |  | 0.003 |
| No | 120 (37.7%) | 88 (27.7%) |  |
| Yes | 44 (13.8%) | 66 (20.8%) |  |
| Adjacent hepatic tissue inflammation, n (%) |  |  | 0.031 |
| None | 72 (30.4%) | 46 (19.4%) |  |
| Mild&Severe | 56 (23.6%) | 63 (26.6%) |  |
| Pathologic stage, n (%) |  |  | 0.044 |
| Stage I&Stage II | 136 (38.9%) | 124 (35.4%) |  |
| Stage III&Stage IV | 36 (10.3%) | 54 (15.4%) |  |
