# Supplementary material for: CGR11 promotes hepatocellular carcinoma progression by regulating autophagy through the PI3K/AKT pathway
Source: Front Cell Dev Biol. 2026 Jan 7;13:1692480. doi: 10.3389/fcell.2025.1692480 (PMC12819743; doi:10.3389/fcell.2025.1692480)
Supplement: Supplementary file 2 [file Image1.pdf]

Supplementary Figures

Fig. S1

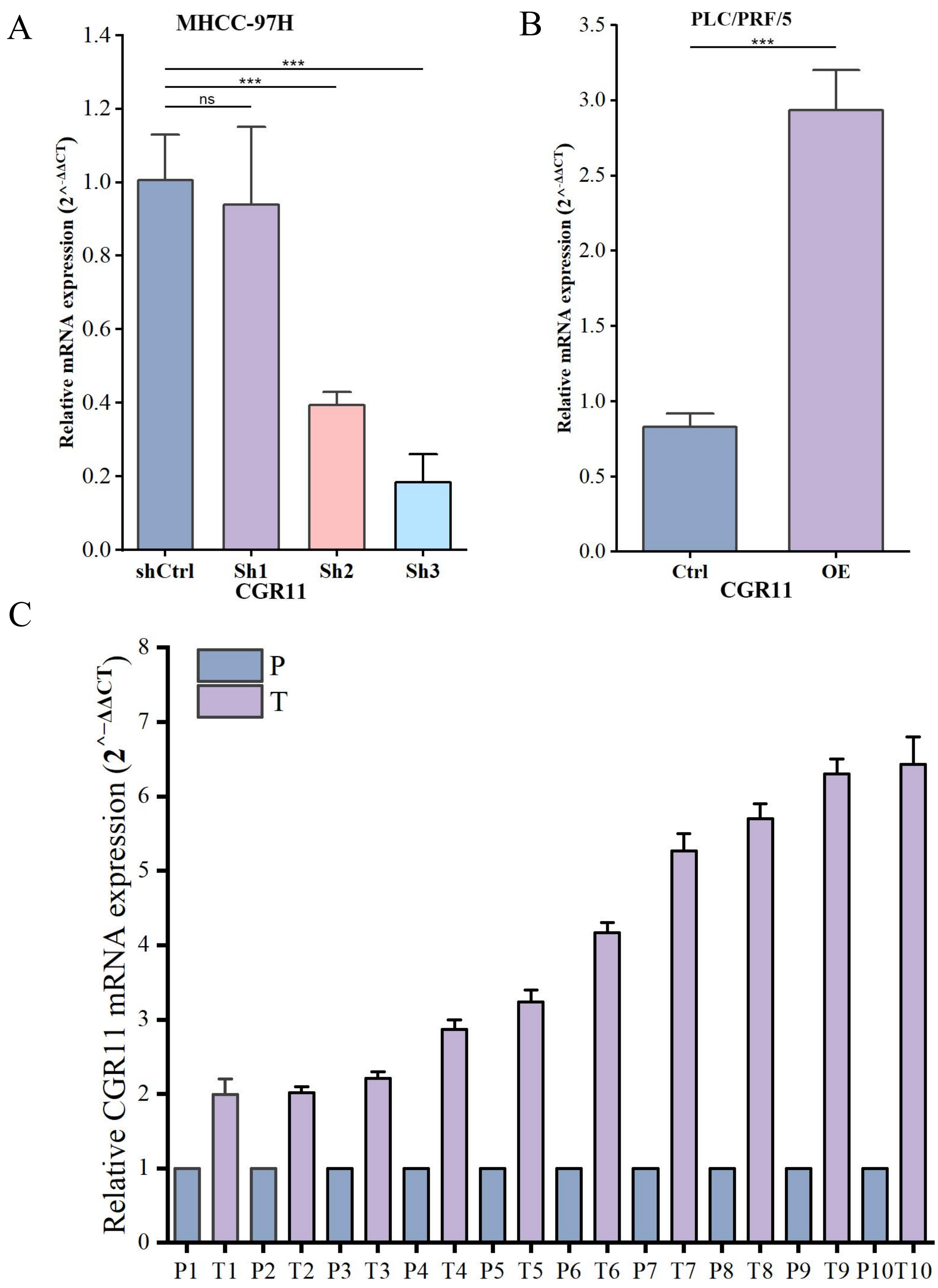

Fig. S1 qRT-PCR showed knockdown/overexpression efficiency of CGR11 in HCC cells were verified and the mRNA level of CGR11 in 10 pairs of representative HCC tissues. (A)qRT-PCR was used to determine the mRNA expression of CGR11 in MHCC-97H^{shCGR11}(three knockdown sequences) and the control cells, (B)the mRNA expression of CGR11 in PLC/PRF/5^{CGR11} and the control cells. (C)qRT-PCR showed the mRNA expression of CGR11 in 10 pairs of representative HCC tissues**.** $**P<0.01$; $***P<0.001$.

Fig. S2

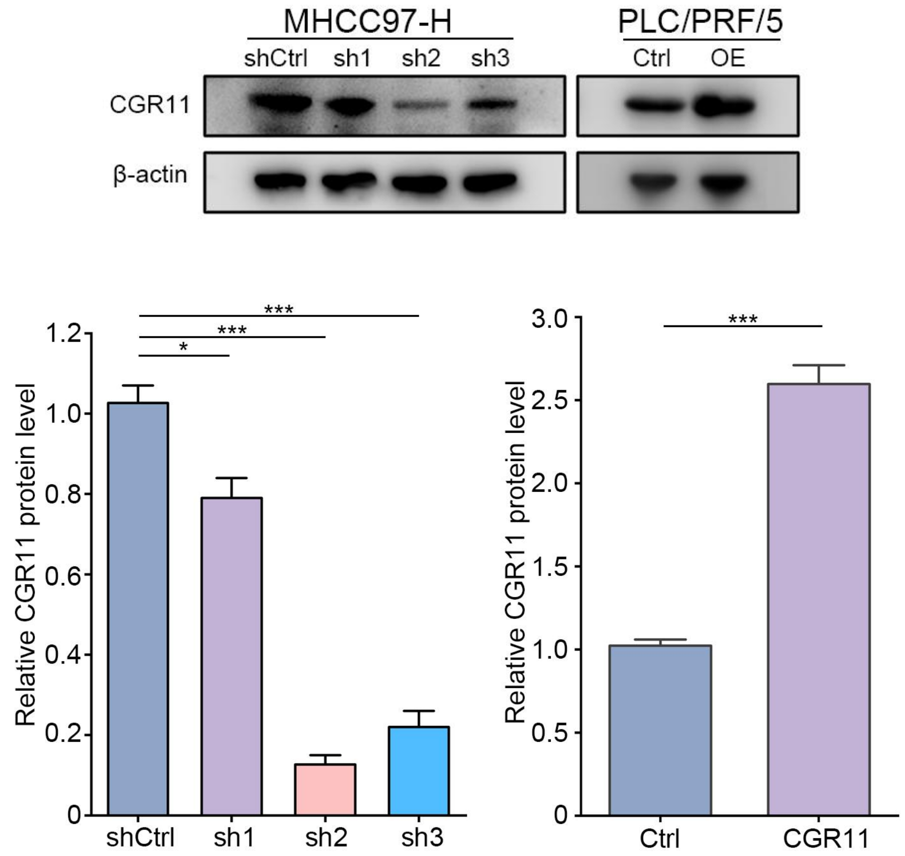

Fig. S2

Western blot verified the overexpression/knockdown efficiency of CGR11 in HCC cells. (A)Western blot was used to determine the protein expression of CGR11 in MHCC-97H shCGR11(three knockdown sequences) and the control cells, (B)the mRNA expression of CGR11 in PLC/PRF/5 CGR11 and the control cells.**P*<0.05, ***P*<0.01, ****P*<0.001.

Fig. S3

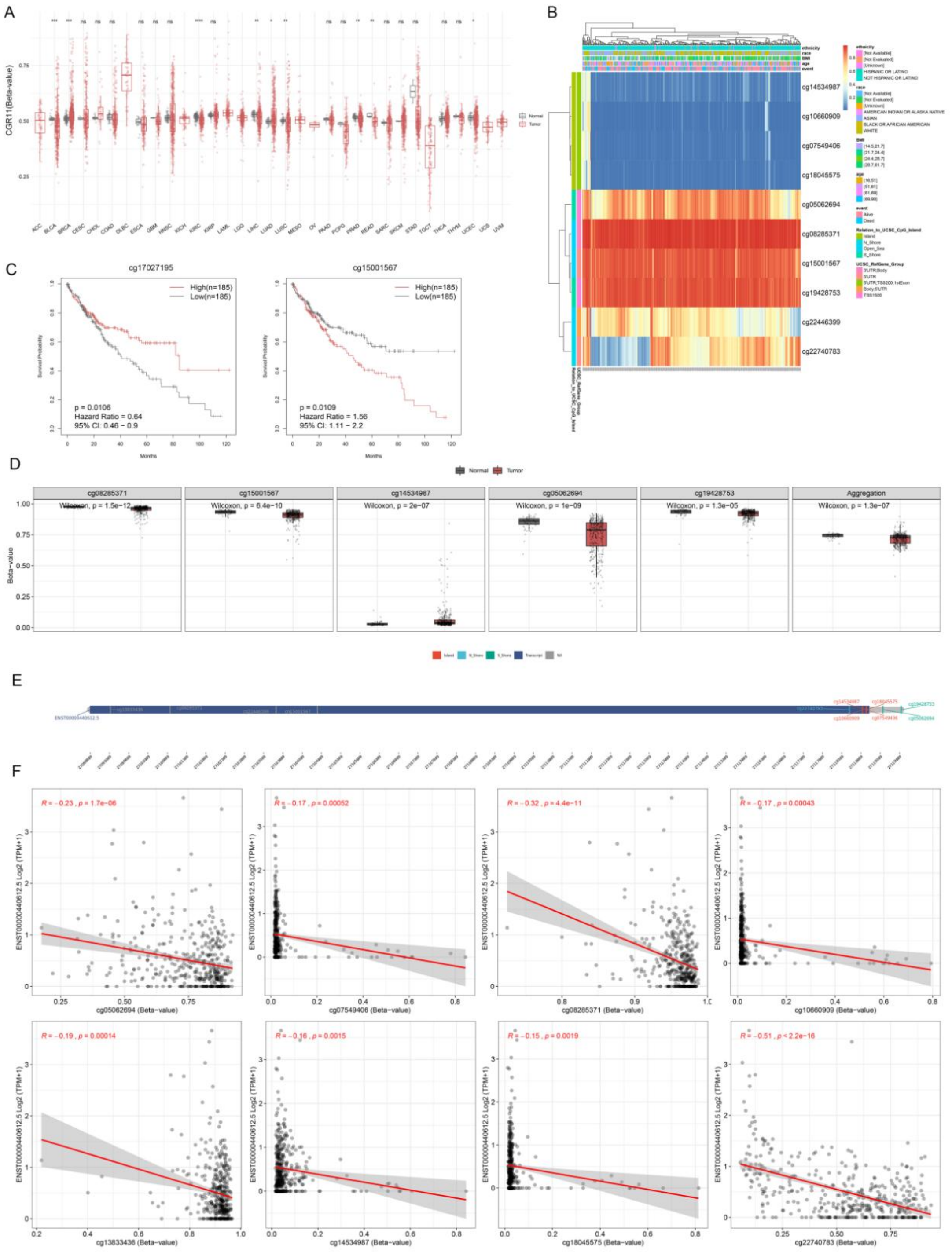

Fig. S3 The DNA methylation of CGR11 in HCC from TCGA data.(A) The methylation levels of CGR11 across tumor tissues and corresponding normal tissues in SMART database. (B) Heatmap integrating DNA methylation of the CGR11 gene in LIHC by MethSurv. (C) Kaplan-Meier survival curves showing two methylation sites in the CGR11 gene. (D)Average methylation levels between normal and tumor tissue stratified by genomic location (Wilcoxon rank sum test) (E)Chromosomal distribution and detailed CpG sites. The promoter region includes six probes (cg14534987; cg19428753; cg05062694; cg07549406; cg18045575; cg10660909). (F) Spearman's correlation between CGR11 expression and methylated sites.

Fig. S4

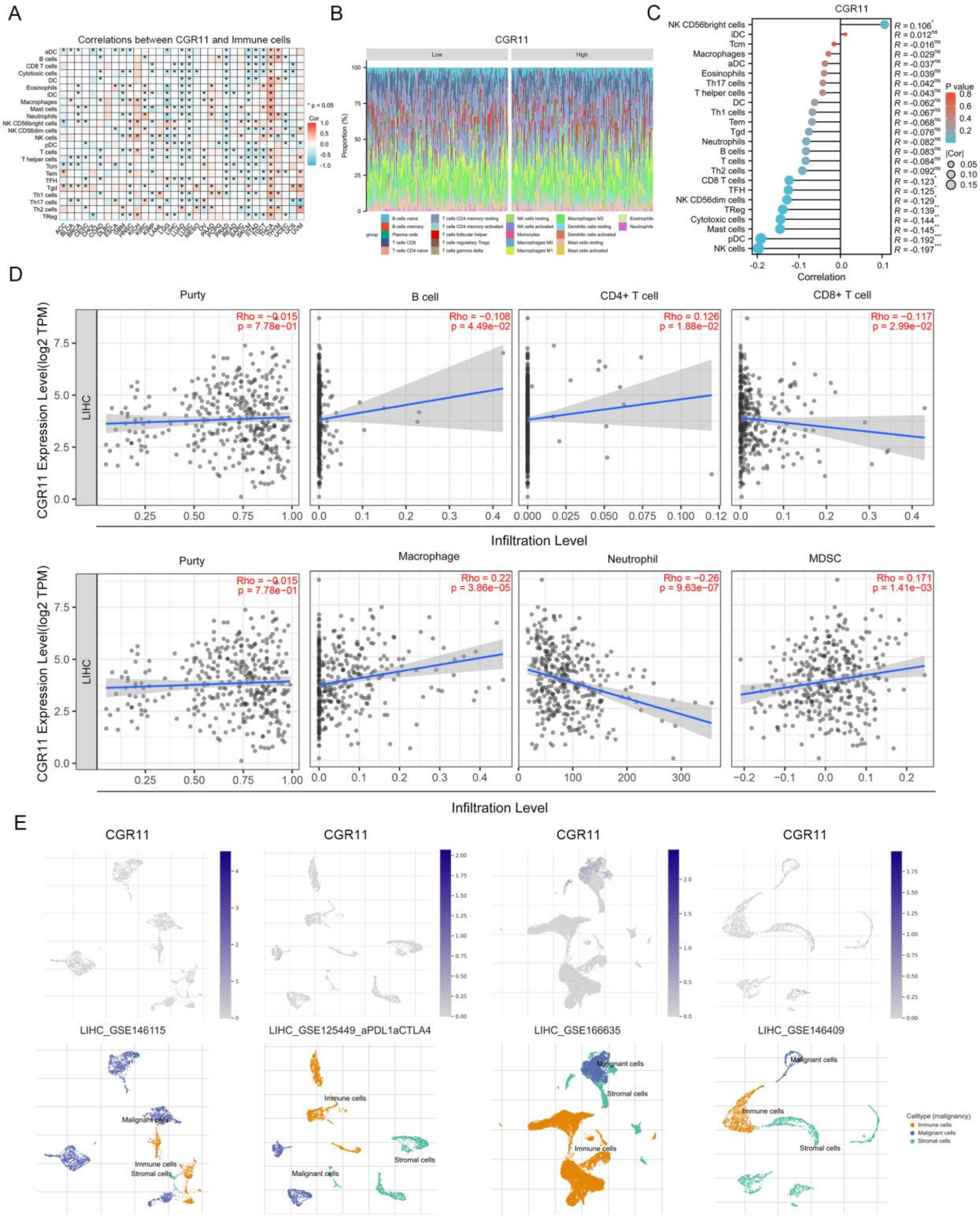

Fig. S4 CGR11-immune interactions through multi-platform integration. (A)Heatmap delineating pan-cancer correlations between CGR11 expression and tumor-infiltrating immune cell composition. (B)Stacked bar chart demonstrating immune cell subset distribution stratified by CGR11 expression levels. (C) Lollipop plot illustrating the correlation between CGR11 expression and immune cell infiltration profiles. (D)Correlation analysis between CGR11 expression and immune infiltrates in LIHC. (E)CGR11 expression in immune cells according to the Gene Expression Omnibus (GEO) GSE146115,GSE125449, GSE166635 and GSE146409 datasets. **P*<0.05, ***P*<0.01, ****P*<0.001.

Fig. S5

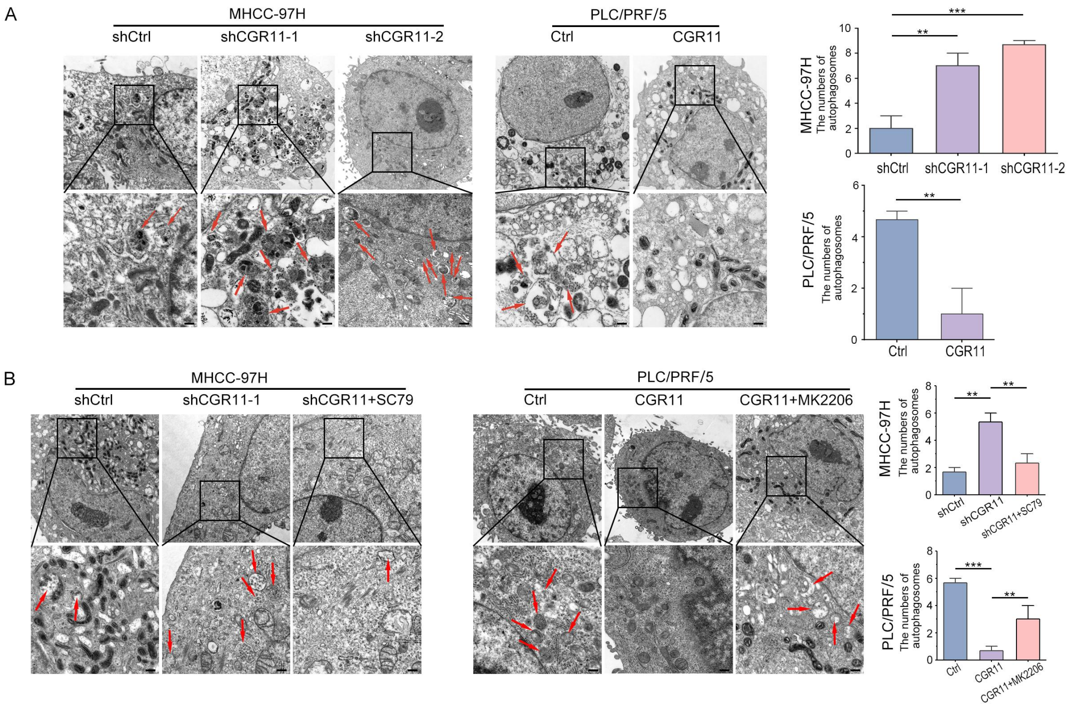

Fig.S5. Quantitative analysis of autophagosomes in the TEM images. (A) Representative transmission electron microscopy images of MHCC-97H^{shCGR11}, PLC/PRF/5^{CGR11} and their control cells. (B) Representative TEM images of MHCC-97H^{shCGR11}, PLC/PRF/5^{CGR11} and their control cells after further treatment with SC79 (4μM) or MK2206 (5μM). Scale bars, 1 μm. $^{**}P < 0.01$; $^{***}P < 0.001$.

Uncropped Western blots

Figure 1

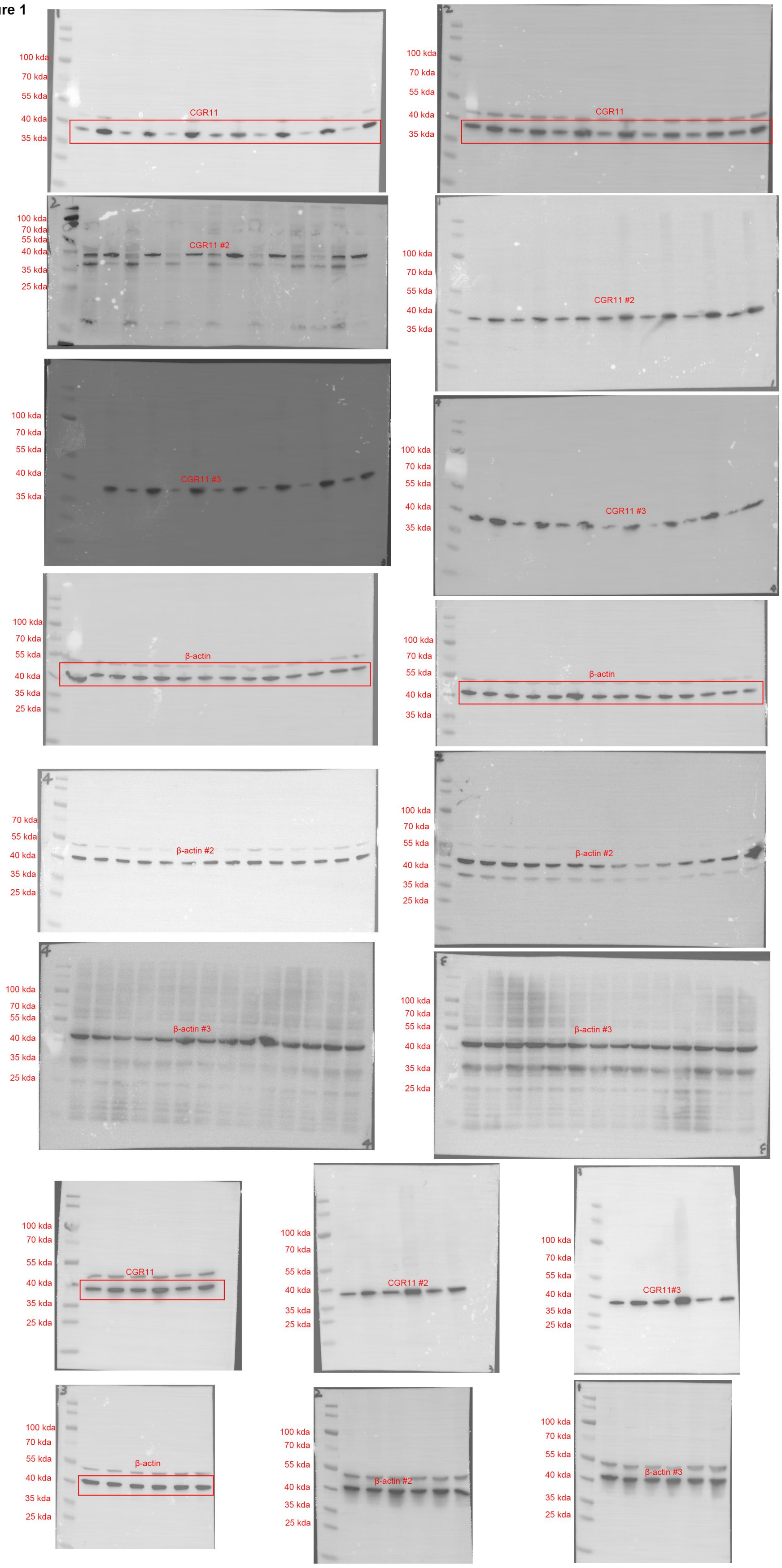

Figure 6

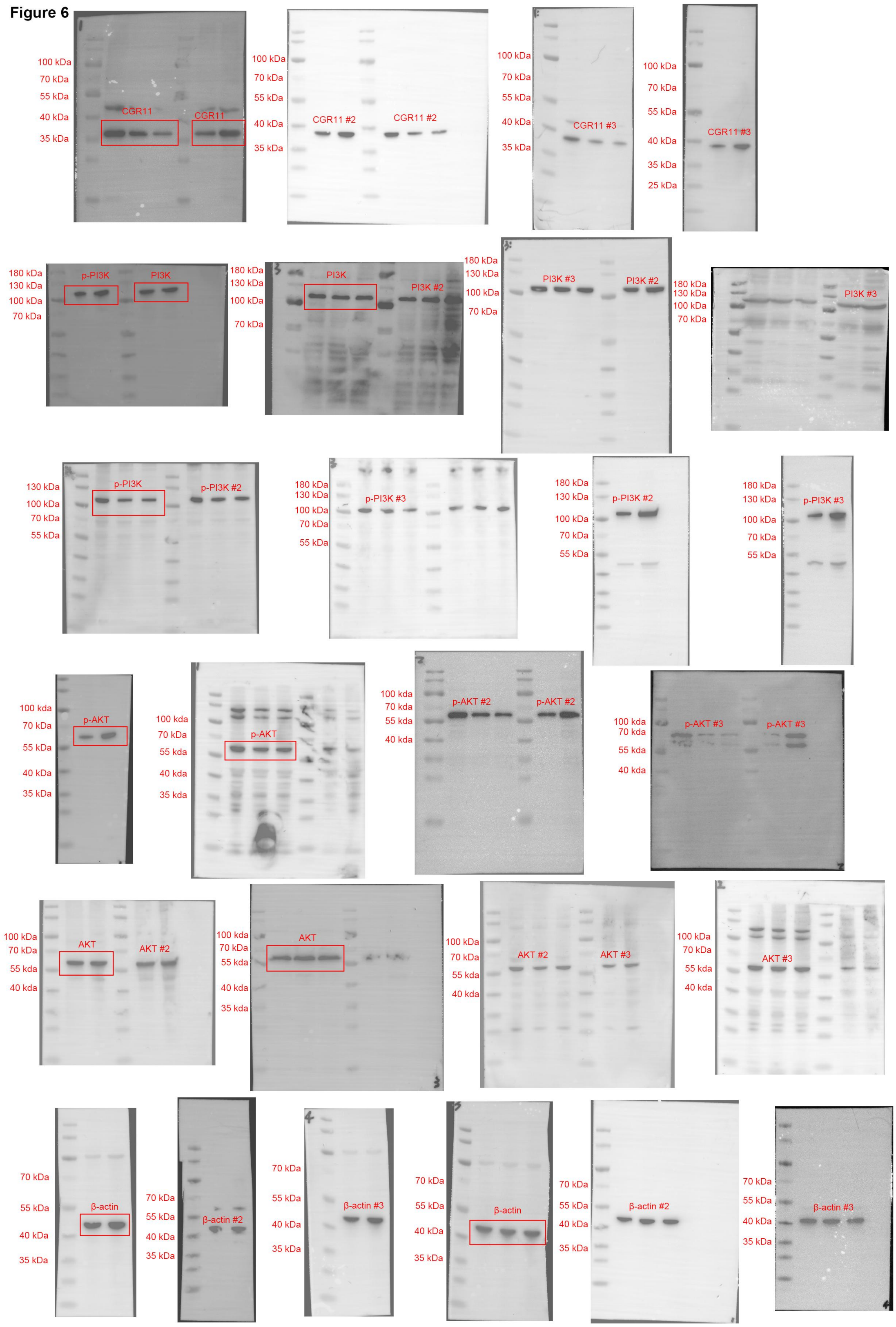
100 kDa
70 kDa
55 kDa
40 kDa
35 kDa
CGR11
CGR11
100 kDa
70 kDa
55 kDa
40 kDa
35 kDa
CGR11 #2
CGR11 #2
100 kDa
70 kDa
55 kDa
40 kDa
35 kDa
CGR11 #3
100 kDa
70 kDa
55 kDa
40 kDa
35 kDa
25 kDa
CGR11 #3
180 kDa
130 kDa
100 kDa
70 kDa
p-PI3K
PI3K
180 kDa
130 kDa
100 kDa
70 kDa
PI3K
PI3K #2
180 kDa
130 kDa
100 kDa
70 kDa
PI3K #3
PI3K #2
180 kDa
130 kDa
100 kDa
70 kDa
PI3K #3
130 kDa
100 kDa
70 kDa
55 kDa
p-PI3K
p-PI3K #2
180 kDa
130 kDa
100 kDa
70 kDa
55 kDa
p-PI3K #3
180 kDa
130 kDa
100 kDa
70 kDa
55 kDa
p-PI3K #2
180 kDa
130 kDa
100 kDa
70 kDa
55 kDa
p-PI3K #3
100 kda
70 kDa
55 kDa
40 kDa
35 kDa
p-AKT
100 kda
70 kda
55 kda
40 kda
35 kda
p-AKT
100 kda
70 kda
55 kda
40 kda
p-AKT #2
p-AKT #2
100 kda
70 kda
55 kda
40 kda
p-AKT #3
p-AKT #3
100 kDa
70 kDa
55 kda
40 kda
AKT
AKT #2
100 kda
70 kda
55 kda
40 kda
35 kda
AKT
100 kda
70 kda
55 kda
40 kda
AKT #2
AKT #3
100 kda
70 kda
55 kda
40 kda
AKT #3
70 kDa
55 kDa
40 kDa
35 kDa
β-actin
70 kDa
55 kDa
40 kDa
35 kDa
β-actin #2
70 kDa
55 kDa
40 kDa
35 kDa
β-actin #3
70 kDa
55 kDa
40 kDa
35 kDa
β-actin
70 kDa
55 kDa
40 kDa
35 kDa
β-actin #2
70 kDa
55 kDa
40 kDa
35 kDa
β-actin #3

Figure 7

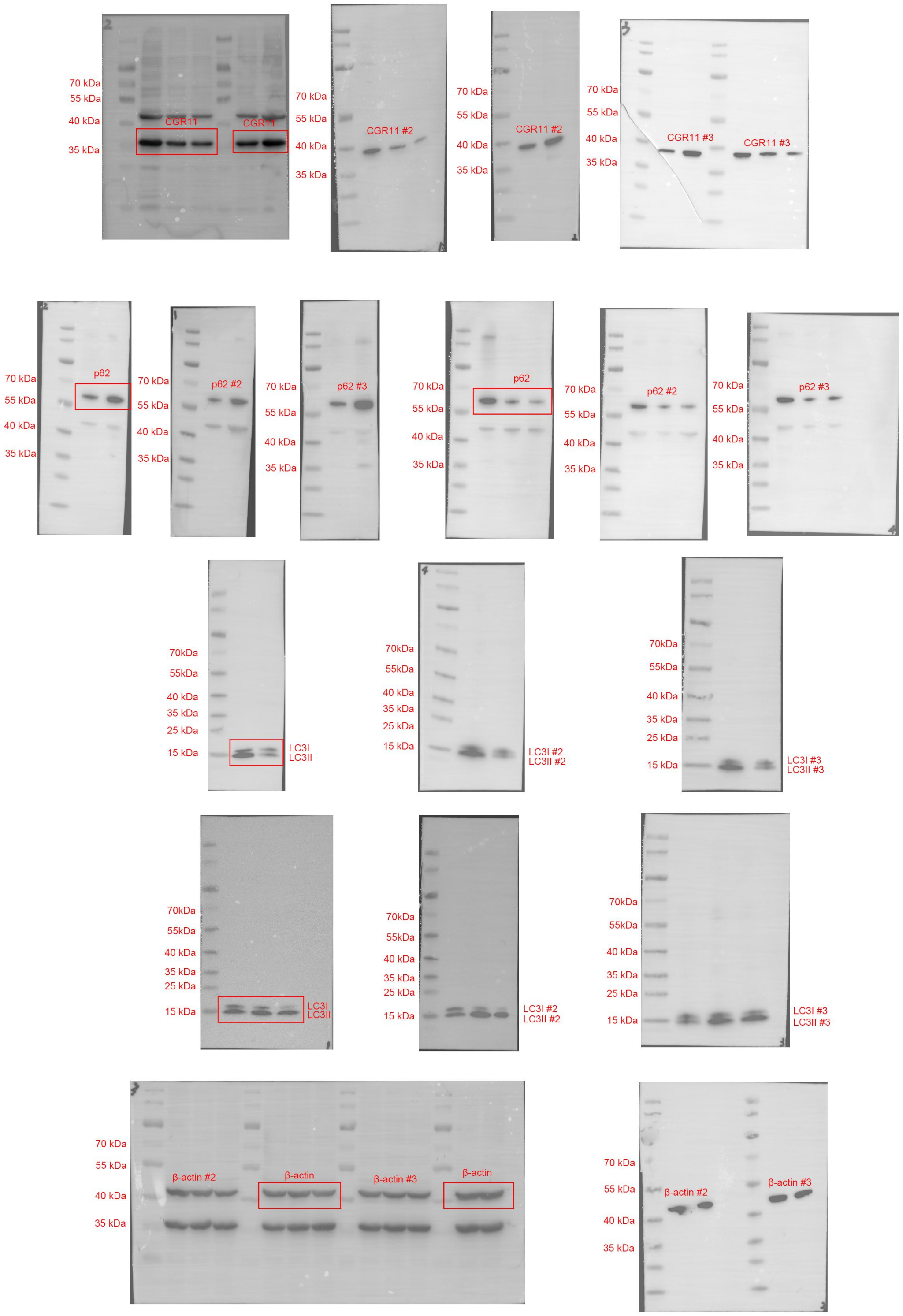

70 kDa
55 kDa
40 kDa
35 kDa
CGR11
CGR11
CGR11 #2
CGR11 #2
CGR11 #3
CGR11 #3
p62
p62 #2
p62 #3
p62
p62 #2
p62 #3
70kDa
55kDa
25 kDa
15 kDa
LC3I
LC3II
LC3I #2
LC3II #2
LC3I #3
LC3II #3
β-actin #2
β-actin
β-actin #3
β-actin
β-actin #2
β-actin #3

Figure 8

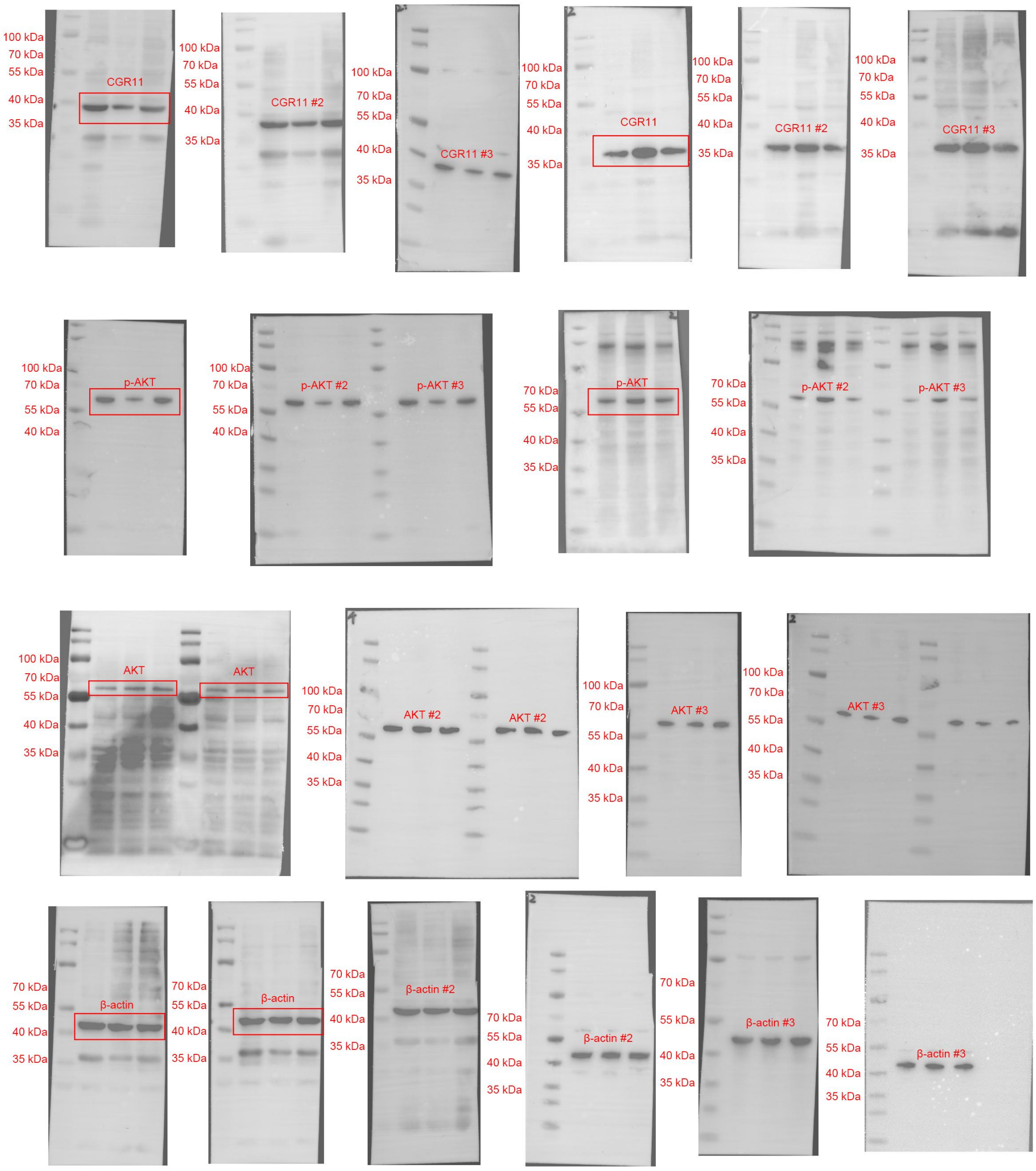
CGR11
CGR11 #2
CGR11 #3
CGR11
CGR11 #2
CGR11 #3
p-AKT
p-AKT #2
p-AKT #3
p-AKT
p-AKT #2
p-AKT #3
AKT
AKT
AKT #2
AKT #2
AKT #3
AKT #3
β-actin
β-actin
β-actin #2
β-actin #2
β-actin #3
β-actin #3
100 kDa
70 kDa
55 kDa
40 kDa
35 kDa

Figure S2

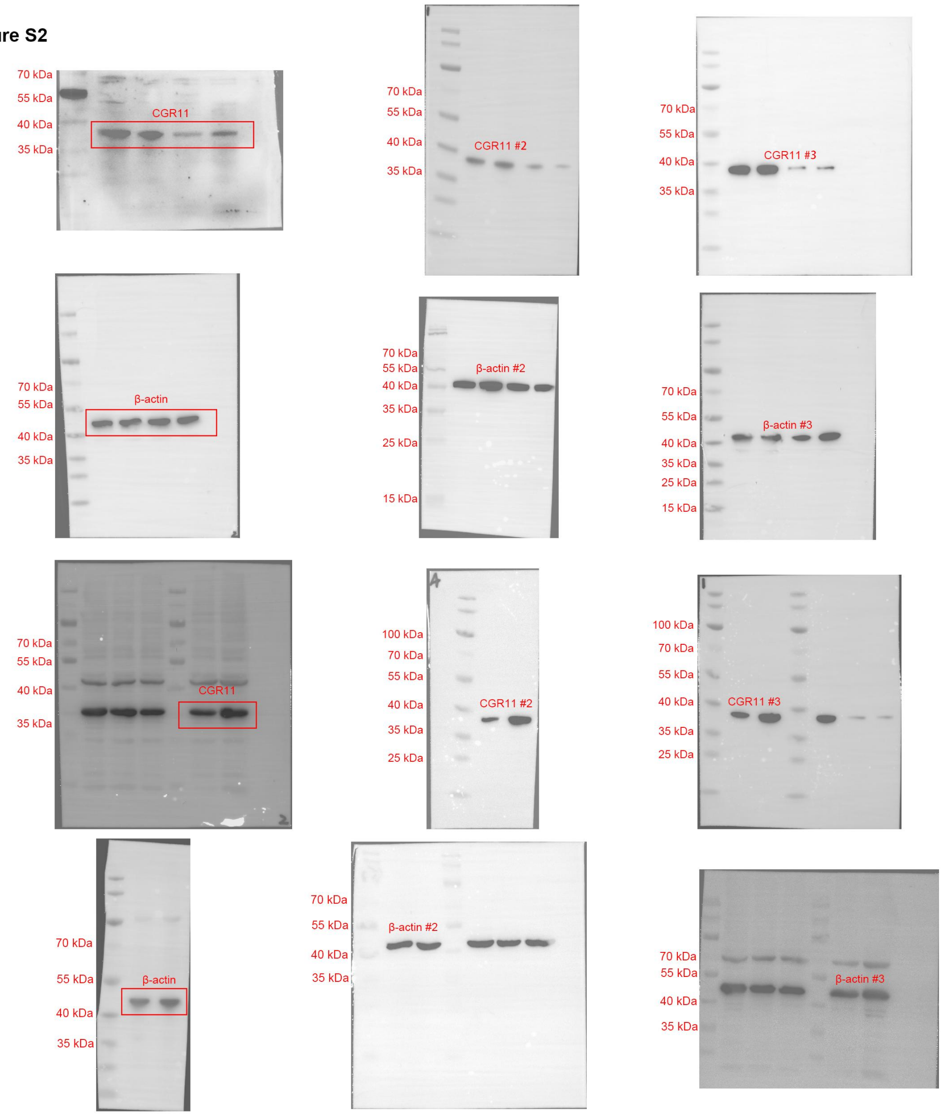
70 kDa
55 kDa
40 kDa
35 kDa
CGR11
70 kDa
55 kDa
40 kDa
35 kDa
CGR11 #2
70 kDa
55 kDa
40 kDa
35 kDa
CGR11 #3
70 kDa
55 kDa
40 kDa
35 kDa
β-actin
70 kDa
55 kDa
40 kDa
35 kDa
25 kDa
15 kDa
β-actin #2
70 kDa
55 kDa
40 kDa
35 kDa
25 kDa
15 kDa
β-actin #3
70 kDa
55 kDa
40 kDa
35 kDa
CGR11
100 kDa
70 kDa
55 kDa
40 kDa
35 kDa
25 kDa
CGR11 #2
100 kDa
70 kDa
55 kDa
40 kDa
35 kDa
25 kDa
CGR11 #3
70 kDa
55 kDa
40 kDa
35 kDa
β-actin
70 kDa
55 kDa
40 kDa
35 kDa
β-actin #2
70 kDa
55 kDa
40 kDa
35 kDa
β-actin #3
